# CFD-DEM coupling analysis of the negative pressure inlet structural parameters on the performance of integrated positive-negative pressure seed-metering device

**DOI:** 10.3389/fpls.2025.1485710

**Published:** 2025-03-14

**Authors:** Dandan Han, Wei Li, Yunxia Wang, Qing Wang, Zhijun Wu, Yuchao Wang, You Xu, Lijia Xu

**Affiliations:** ^1^ College of Mechanical and Electrical Engineering, Sichuan Agricultural University, Ya’an, China; ^2^ Nanjing Institute of Agricultural Mechanization, Ministry of Agriculture and Rural Affairs, Nanjing, China

**Keywords:** maize, seed-metering device, integrated positive-negative pressure, air inlet, CFD-DEM coupling approach

## Abstract

To assess the influence of the structural parameters of the negative pressure inlet pipe on the seeding performance of the integrated positive-negative pressure seed-metering device, angles *α* and *β*, along with taper *θ*, were selected as test variables for conducting coupling simulation tests and physical bench tests. The results indicate that the average differential pressure across the holes in the seed-filling zone (Δ*p_sf_*), the airflow rate at the interaction interface between the negative pressure inlet pipe and the negative pressure chamber (*Q*), the average drag force on seeds in the seed-filling zone (*F*_D,_ *_sf_*), and the average drag force on seeds in the seed-cleaning zone (*F*_D,_ *_sc_*) are all significantly affected by the test factors. The structural parameters of the negative pressure inlet pipe can be accurately predicted using the prediction model developed through regression analysis of the central composite test results. The optimal parameters combination is established as 17.1° for angle *α*, 81.3° for angle *β*, and 0.52° for taper *θ*. The maximum error between the predicted evaluation index values and those obtained from the coupling simulation verification test with the optimized negative pressure inlet pipe is less than 6.66%, indicating a strong correlation and reasonable prediction of the structural parameters. The results from the physical bench tests confirm that the optimal operational speed for the seed-metering device with the optimized negative pressure inlet pipe is 4 to 5 km/h, with an optimal working negative pressure of 4 to 6 kPa. Under these conditions, the qualified rate varies from 92.27% to 95.28%, the multiple rate from 2.43% to 5.52%, and the leakage rate from 1.22% to 5.2%.

## 1 Introduction

The soybean-maize banded composite planting technology represents a viable strategy for stabilizing maize yields while simultaneously expanding soybean cultivation, thereby providing a novel technical approach to bolster China’s maize production capacity and enhance soybean self-sufficiency rates (Yang and Yang, 2019). A fundamental aspect of this planting pattern involves minimizing the spacing between maize and soybean seeds during sowing (Yang et al., 2022; Zhang et al., 2020). To address the demand for precision sowing in densely planted conditions, the development of compatible precision seed-metering devices is essential (Karayel and Özmerzi, 2001; Vianna et al., 2014; Wang et al., 2021).

The air-suction type serves as a pneumatic seed-metering device, offering numerous advantages such as improved seed spacing accuracy, reduced seed damage, and adaptability to a diverse array of seeds with only minor modifications to the device (Shafii and Holmes, 1990; Guarella et al., 1996; Karayel et al., 2022). Jack et al. (2013) identified a correlation between the total area of the vacuum holes and the operational efficiency of the air-suction seed-metering device, determining that optimal seeding performance occurs within the 400~650 mm^2^ range when examining the interplay of rotational speed and pressure. To enhance the seeding efficacy of air-suction seed-metering devices at elevated operating speeds in densely planted scenarios, various researchers have explored the effects of seeding plate design on seed population disturbance and mobility (Wang et al., 2020; Shi et al., 2020; Liu et al., 2022a).

Kostić et al. employed both theoretical and experimental methodologies to investigate the causes of maize seeding failures, revealing that observable variables significantly impact seeding distribution accuracy (Kostić et al., 2018). During the validation of seeding performance, high-speed cameras have proven to be effective tools for replicating the dynamic processes and capturing the movement of seeds within the seed-metering device, surpassing the observational capabilities of the human eye (Pezzuolo et al., 2018; Tang et al., 2023; Zhao et al., 2024). The operation of the air-suction seed-metering device involves continuous collisions and friction among seeds and between seeds and the device, resulting in highly complex mechanical behaviors during seed movement that are challenging to analyze as previously described (Lai et al., 2016; Pareek et al., 2023).

To further improve seeding performance, a positive pressure inlet pipe was integrated into the seed-casting zone of the air-suction seed-metering device designed in the previous stage (Han et al., 2024). This modification aimed to facilitate seed voting, resulting in a positive-negative pressure combined seed-metering device. The addition of the positive pressure inlet pipe and chamber altered the operational area of the negative pressure chamber, thereby influencing the uniformity of pressure within the airflow field and enhancing the adsorption effect. The uniformity of the airflow pressure within the negative pressure chamber is directly affected by the structural parameters of the negative pressure inlet pipe, necessitating an optimization of these parameters.

A standard gas-solid two-phase flow coupling field, comprising an airflow field and a seed particle field, operates inside the seed-metering device (Ei-Emam et al., 2021). This analysis not only provides a comprehensive understanding of the key variables affecting the operational efficiency of the seed-metering device and establishes a reasonable range for the working parameters, but it also mitigates the uncertainty associated with prototype trial production through the application of CFD-DEM coupling simulations (Han et al., 2018; Liu et al., 2022b; Darabi et al., 2011). Consequently, a simulation test to assess the influence of the structural parameters of the negative pressure inlet pipe on seeding performance was conducted utilizing the CFD-DEM coupling simulation and analysis methodology in this study. To identify the ideal operating parameters for the seed-metering device equipped with the optimized negative pressure inlet pipe, the adaptability to various operational conditions was evaluated.

## 2 Structure and working principle of the seed-metering device

### 2.1 Structure of the seed-metering device

**Figure 1:**
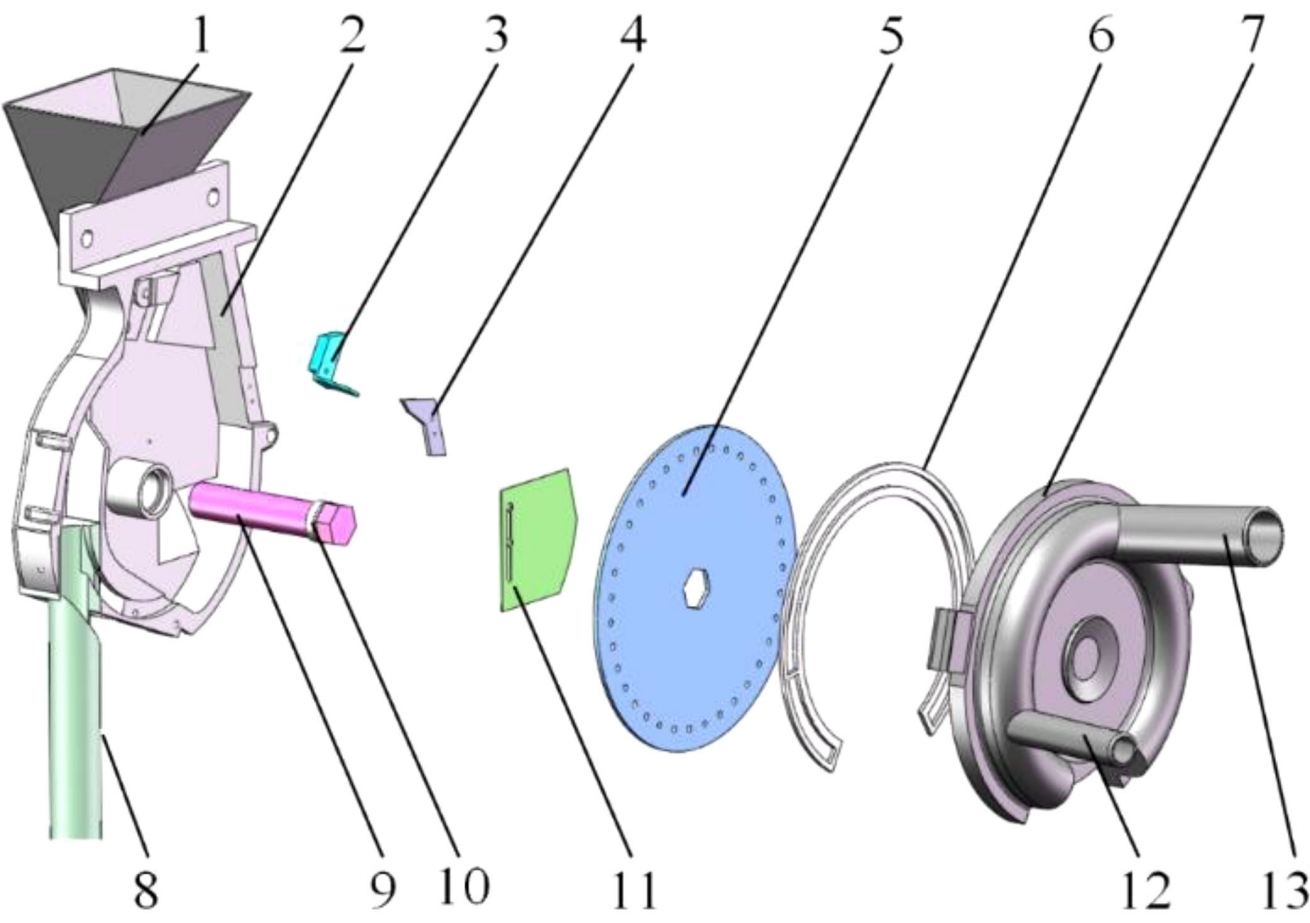
Structural diagram of the positive-negative pressure combined seed-metering device. 1. Seed inlet; 2. Front shell; 3. Internal cleaning blade; 4. External cleaning blade; 5. Seeding plate; 6. Sealing ring; 7. Rear shell; 8. Seeding tube; 9. Seeding shaft; 10. Bearing; 11. Seed layer height adjustment plate; 12. Positive pressure inlet pipe; 13. Negative pressure inlet pipe.

The structural schematic of the positive-negative pressure combined maize precision seed-metering device designed in this study is depicted in **Figure 1**. It primarily consists of a front shell, internal and external cleaning blades, a seeding plate, positive and negative pressure inlet pipes, a seed layer height adjustment plate, a rear shell, and a seeding tube, among other components. The internal and external cleaning blades are positioned adjacent to the seeding plate to eliminate extraneous seeds near the holes, guaranteeing a consistent single seeding rate. The seed layer height adjustment plate is situated beneath the seed inlet to regulate and control the height of the seed pile. A sealing ring is integrated between the negative pressure chamber and the seeding plate to maintain the integrity of the negative pressure chamber.

**Figure 2:**
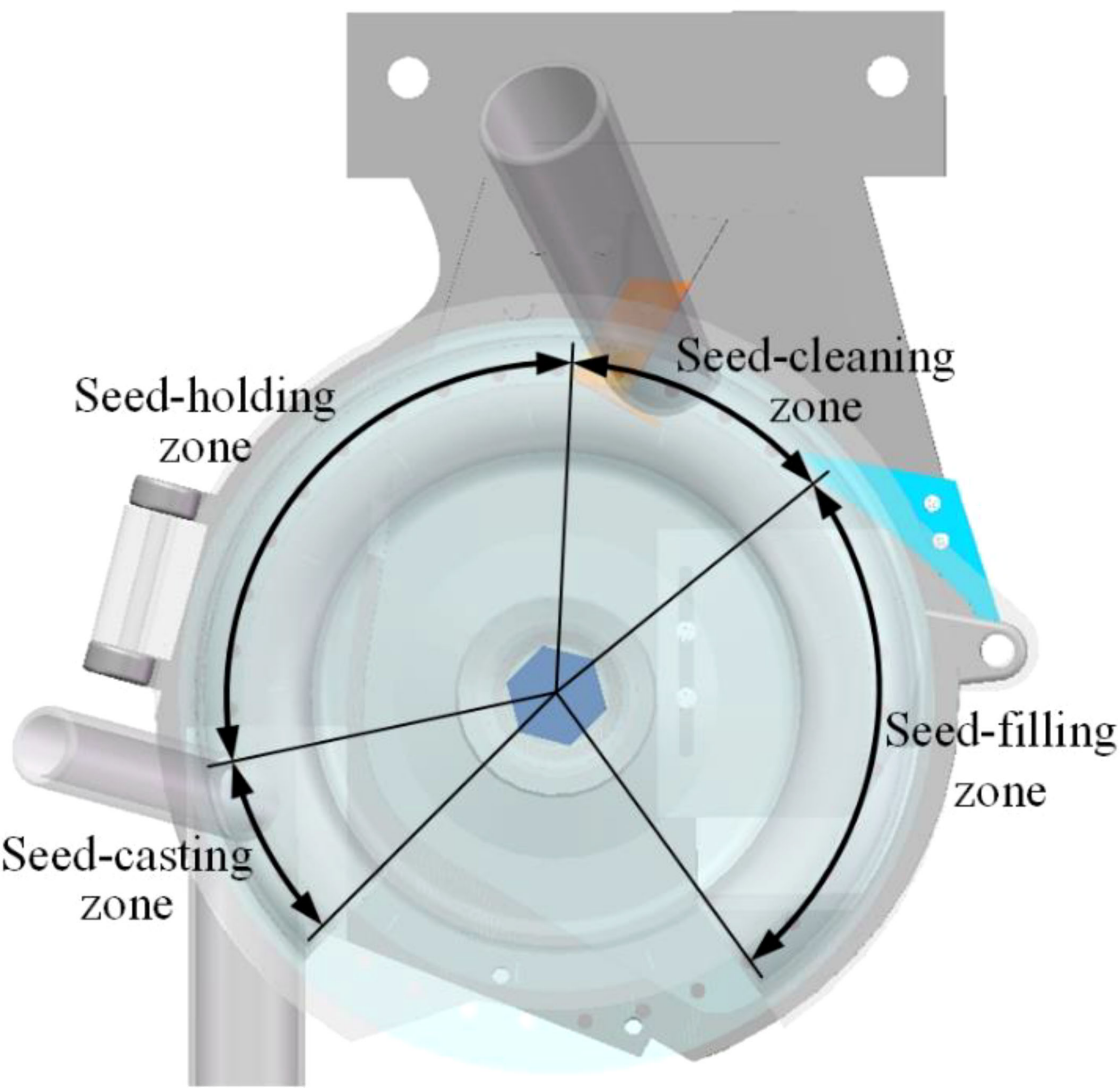
Schematic illustration of the seed-metering device’s working process.

The working principle of the seed-metering device can be delineated into four distinct stages: filling, cleaning, holding, and casting. The respective zones traversed by the seeds include the seed-filling, seed-cleaning, seed-holding, and seed-casting zones, as illustrated in **Figure 2**. The seed-filling zone extends from the base of the seed-filling chamber to the external cleaning blade, connected by a 90° arc that links the working zones. The seed-cleaning zone is defined as the area between the internal and external cleaning blades, featuring a working arc of approximately 45°. The seed-holding zone spans from the internal cleaning blade to the terminus of the negative pressure chamber, with a working interval of 110°. The positive pressure chamber encompasses the seed-casting zone, characterized by a 35° working interval arc.

When the seed-metering device is operational, seeds are introduced into the seed-filling zone via the seed inlet, while the seed layer height adjustment plate ensures the maintenance of the appropriate seed layer. As the seeding plate rotates counterclockwise, seeds located near the holes in the seed-filling area are drawn into the holes by the adsorption force generated by the negative pressure chamber. The internal and external cleaning blades progressively eliminate excess seeds as the adsorbed seeds transition to the seed-cleaning zone, leaving only one seed that remains stably adsorbed. The holes containing the adsorbed seeds gradually rotate from the seed-holding zone into the seed-casting zone. At this juncture, the air pressure on both sides of the hole shifts from negative to positive, causing the seed to transition from an adsorption state to a blowing. Consequently, the seed that has lost its adsorption force will fall from the hole due to the combined effects of gravity and centrifugal force, subsequently entering the seed guide pipe.

### 2.2 Kinematic properties of seeds in the seed-metering device

The seeding performance is ultimately dictated by the movement dynamics of the seed. Therefore, analyzing the movement characteristics of the seeds not only offers a more comprehensive and intuitive understanding of the working principle but also identifies the factors that directly influence seeding performance through the examination of the seeds’ movement process. This analysis enables to exploration of the relationship between structural parameters, operational parameters, and the influencing factors. Thus, investigating the movement of seeds within the seed-metering device proves to be highly beneficial.

#### 2.2.1 Force analysis of the seed-filling process

**Figure 3:**
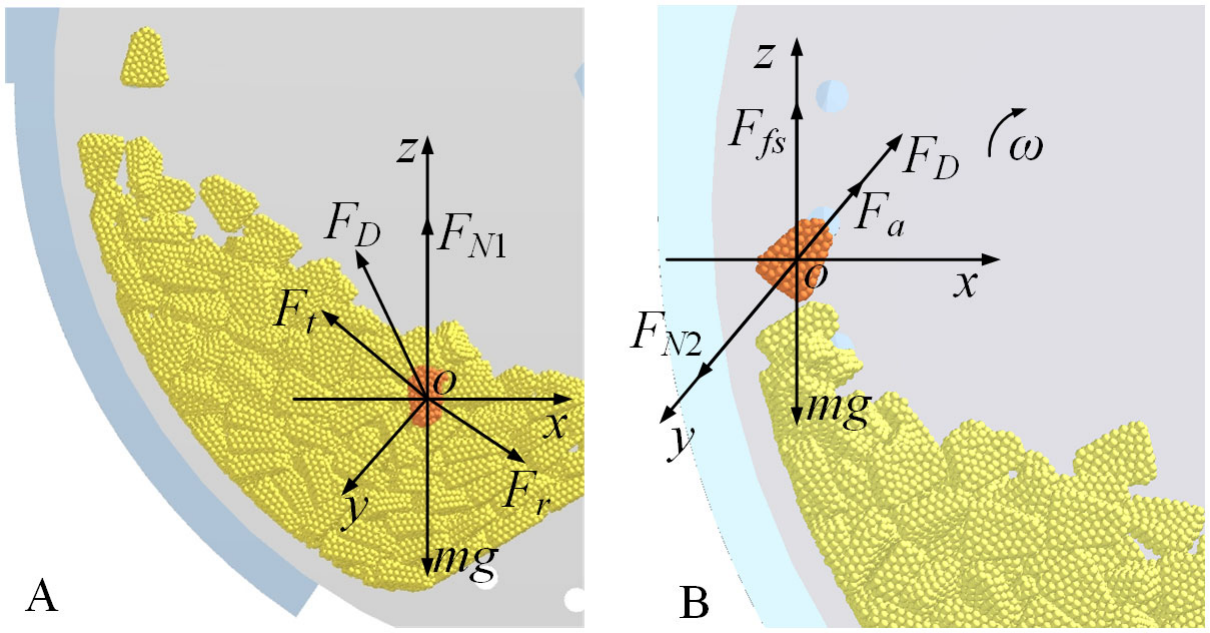
Force analysis of seeds during the seed-filling process **(A)** is the seed in the seed pile stage; **(B)** is the seed adsorbed to the hole.

The seed-filling process is divided into two distinct phases to examine the forces acting on the seed, with a spatial coordinate system established using the seed’s center as the origin, as depicted in **Figure 3**. In this spatial coordinate system, the horizontal direction is designated as the x-axis, the direction perpendicular to the seeding plate is the y-axis, and the vertical direction is represented by the z-axis. When the seed is positioned within the seed layer, it experiences the turbulent force (*F_t_*) from the turbulent seed cam, in addition to its gravitational force. The seeds tend to migrate in the same direction as the rotation of the seeding plate. During the seed-filling process, seeds are subjected to airflow drag force (*F_D_*), gradually moving closer to the designated hole. However, they also encounter friction and crowding pressure from the surrounding population, which hinders their adsorption process. Once the seed overcomes the total resistance force (*F_r_*) from the population and becomes adsorbed to the hole, it will then experience the adsorption force (*F_a_*).

Based on the force state of the seed within the seed pile, the condition necessary for the seed to detach from the seed pile and advance toward the hole is established as Equation 1.


(1) $$\left\{ \begin{array}{l} \sum F_{x}\geq 0:F_{D}\cos\varphi_{Dx}+F_{t}cos\varphi_{tx}\geq F_{r}\cos\varphi_{rx}\\ \sum F_{y}\geq 0:F_{D}\cos\varphi_{Dy}+F_{t}cos\varphi_{ty}\geq F_{r}\cos\varphi_{ry}\\ \sum F_{z}\geq 0:F_{N1}+F_{D}\cos\varphi_{Dz}+F_{t}cos\varphi_{tz}\geq mg+F_{r}\cos\varphi_{rz} \end{array}\right.$$

Where *F_D_* is the drag force in N, *φ_Dx_*, *φ_Dy_*, and *φ_Dz_* are the angles of *F_D_* with respect to the x-axis, y-axis, and z-axis in (°), *F_t_* is the turbulent force in N, *φ_tx_*, *φ_ty_*, and *φ_tz_* are the angles of *F_t_* relative to the x-axis, y-axis, and z-axis in (°), *F_r_* is the total resistance force acting on the seed during the seed-filling process in N, *φ_rx_*, *φ_ry_*, and *φ_rz_* are the angles of *F_r_* concerning the x-axis, y-axis, and z-axis in (°), *F_N_*_1_ is the support force exerted on the seed by the seed pile in N, *m* is the mass of the seed in kg, and *g* is the gravitational acceleration in m/s^2^.

The drag force (*F_D_*) varies with the pressure exerted in the negative pressure chamber. The drag force can be estimated using Equation 2 provided below (Han et al., 2017; Su et al., 2023):


(2) $$F_{D}=\frac{1}{2}\rho_{g}A_{p}C_{D}|v_{g}-v_{p}|(v_{g}-v_{p})$$

Where *ρ_g_* is the gas density in kg/m^3^, *A_p_* is the windward area of the particle in m^2^, *C_D_* is the drag force coefficient, *v_g_* is the gas velocity in m/s, and *v_p_* is the particle velocity in m/s.

A Rayleigh curve plotted against the Reynolds number (Re*_p_*) (*C_D_* =*f* (Re*_p_*) can be utilized using Equation 3 to determine the aerodynamic drag coefficient (*C_D_*) of the particles (Mudarisov et al., 2017).


(3) CD={24Rep,(Rep≤0.5)24(1.0+0.25Rep0.687)Rep,(0.5<Rep≤1000)0.44,(Rep>1000)

In this scenario, the Reynolds number of the particle is calculated using Equation 4.


(4) Rep=ρgdp(vg−vp)μ

Where *d_p_* is the equivalent diameter of the particles in m, and *μ* is the dynamic viscosity of air in PA·s (at 20°C, *μ*=1.82×10^−5^ PA·s).

Upon detachment from the adhering population and stabilization within the hole, the forces reach equilibrium that is shown in Equation 5:


(5) $$\left\{ \begin{array}{l} \sum F_{y}=0:F_{D}+F_{a}-F_{N2}=0\\ \sum F_{z}=0:F_{fs}-mg=0 \end{array}\right.$$

Where *F_a_* is the adsorption force of the seed by the hole in N, *F_N_*_2_ is the support force exerted by the seeding plate in N, and *F*_fs_ is the frictional force between the seed and the seeding plate in N.

The force analysis during the seed-filling process reveals that an increase in both the drag force (*F_D_*) and the adsorption force (*F_a_*) enhances the seed-filling efficiency. The adsorption force is directly proportional to the differential pressure across two sides of the hole.

#### 2.2.2 Force analysis of the seed-cleaning process

**Figure 4:**
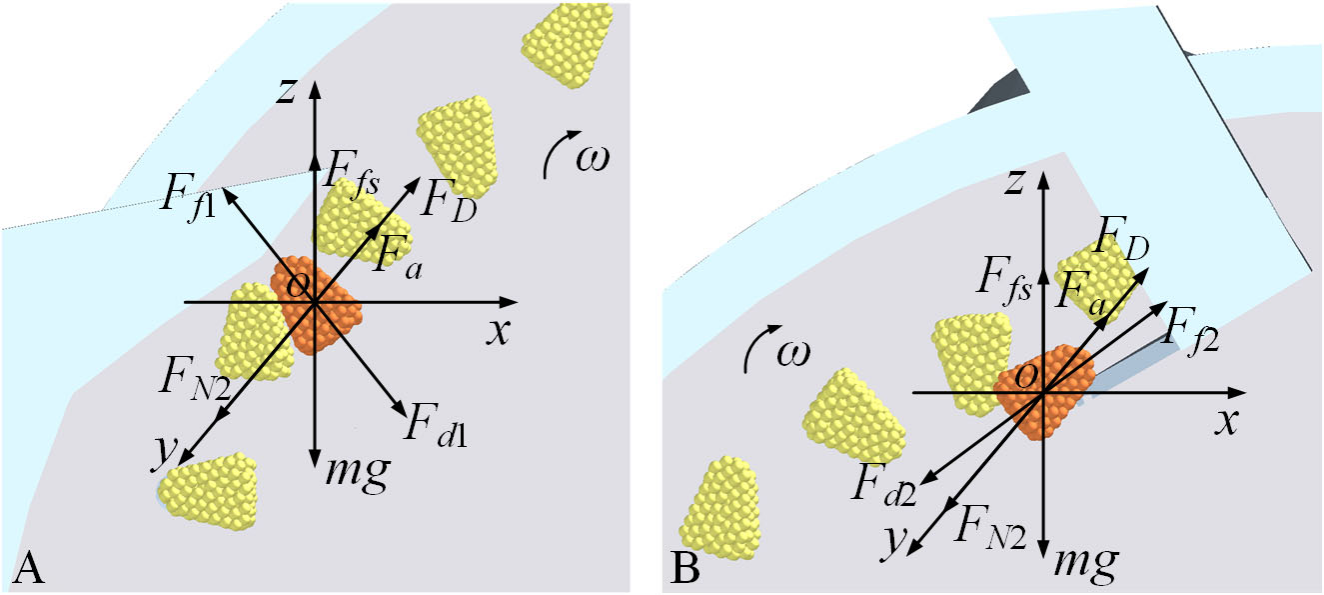
Force analysis of seeds during the seed-cleaning process **(A)** is the outer seed-cleaning zone; **(B)** is the inner seed-cleaning zone.

**Figure 4** depicts the findings of the force analysis on seeds within both the internal and external seed-cleaning zones. As the seeds are rotated into these zones by the seeding plate, they experience the airflow drag force *F_D_*, the support force from the seeding plate *F_N_*_2_, the driving forces from the internal and external seed-cleaning blades *F_d_*_2_ and *F_d_*_1_, the frictional forces between the seed and both the seeding plate and the seed-cleaning blades, as well as the adsorption force from the holes *F_a_*, in addition to the gravitational force acting on the seeds.

Seeds in an unstable state near the holes are dislodged by the action of the external and internal seed-cleaning blades, as exemplified by the forces acting on the seed (Equation 6) during the cleaning process of the external seed-cleaning blade:


(6) $$\left\{ \begin{array}{l} \sum F_{x}\geq 0:F_{d1}cos\varphi_{dx}\geq F_{f1}\cos\varphi_{fx}\\ \sum F_{y}=0:F_{D}+F_{a}-F_{N2}=0\\ \sum F_{z}\geq 0:mg+F_{d1}cos\varphi_{dz}-F_{fs}-F_{f1}\cos\varphi_{fz}\geq 0 \end{array}\right.$$

Where *F_d_*_1_ is the driving force of the external seed-cleaning blade in N, *φ_dx_* and *φ_dz_* are the angles of *F_d_*_1_ with respect to the x-axis and z-axis in (°), *F*_f1_ is the frictional force between the seed and the external seed-cleaning blade in N, *φ_fx_* and *φ_fz_* are the angles of *F*_f1_ concerning the x-axis and z-axis in (°).

The force analysis conducted during the seed-cleaning process, as illustrated in **Figure 4**, indicates that an increase in the drag force (*F_D_*) acting on the seed correlates with improved seed-filling efficiency. It is crucial to maintain the adsorption force within the seed-cleaning zone, or differential pressure, at an optimal level. The excessive force may hinder the effectiveness of the seed-cleaning function.

#### 2.2.3 Force analysis of the seed-holding process

**Figure 5:**
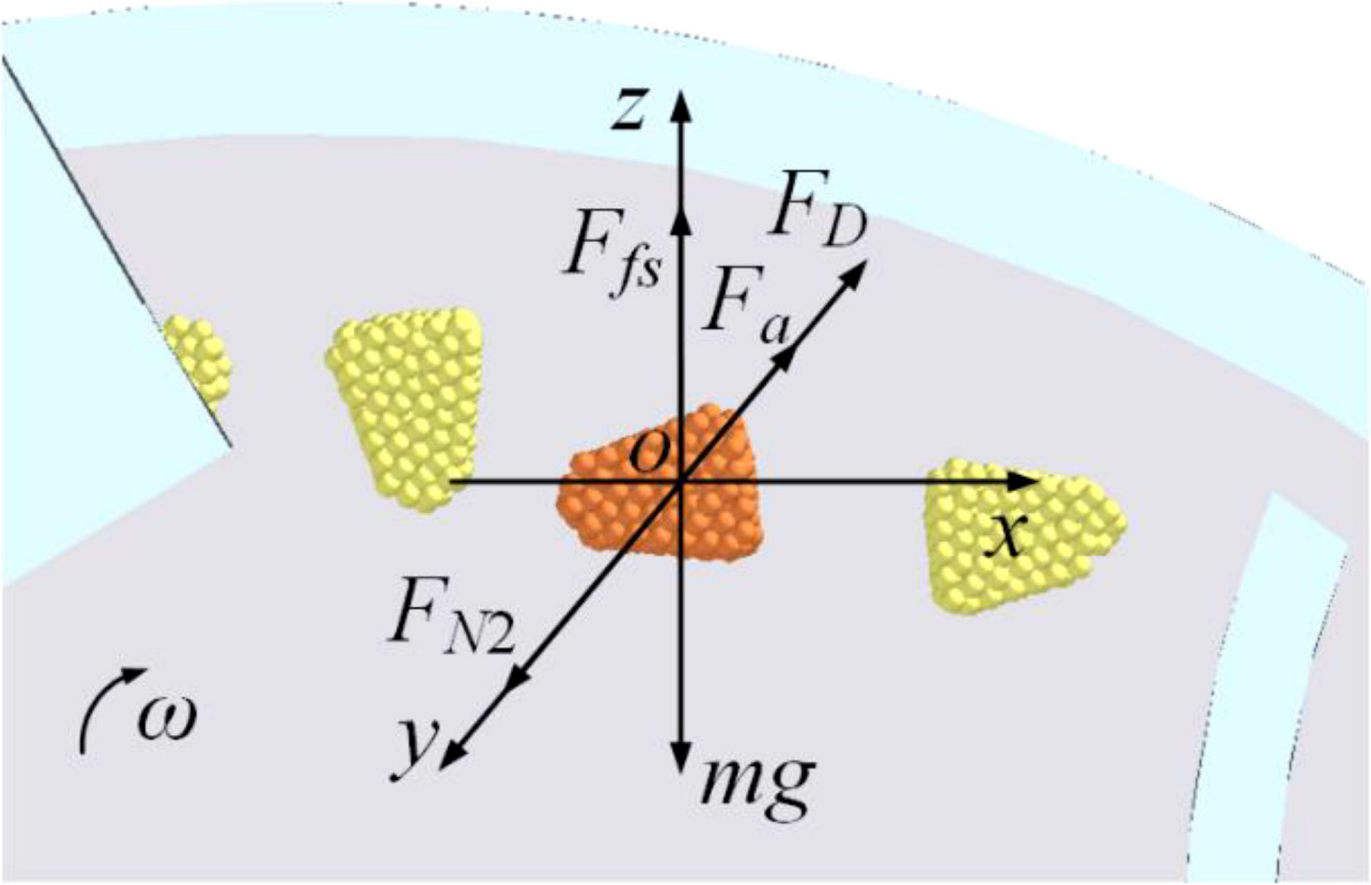
Force analysis of seeds during the seed-holding process.

The results of the force analysis on the seeds within the seed-holding zone are depicted in **Figure 5**. During the seed-holding process, the seed experiences identical forces as illustrated in **Figure 3b** when it is separated from the population and adheres to the hole. The force analysis can be referenced in Equation 5.

The force analysis of the seed during the seed-holding process reveals that an increase in both the drag force (*F_D_*) and the adsorption force (*F_a_*) enhances the seed-holding capability.

### 2.3 Structural optimization of the negative-pressure air inlet

The positive-negative pressure combination seed-metering device primarily facilitates the adhesion of seeds to the seeding plate through the differential pressure generated by the negative-pressure chamber flanking the perforations. A more constant pressure within the airflow field significantly contributes to improved seeding uniformity and reduced leakage rate (Ding et al., 2018). The structural and positional features of the negative-pressure inlet pipe directly influence the pressure homogeneity of the airflow field across the chamber, thereby affecting overall seeding performance. Thus, optimizing the structural parameters of the negative pressure inlet pipe is critical for enhancing seeding efficacy. The force analysis of seeds across various working areas, as discussed in section 2.2, indicates that the direction of incoming airflow plays a pivotal role in the seeds’ adsorption capacity. The negative pressure inlet pipe is strategically positioned at the center of the negative pressure chamber to ensure a consistent adsorption force for each perforation, as a larger area within the negative pressure chamber necessitates a greater negative pressure.

**Figure 6:**
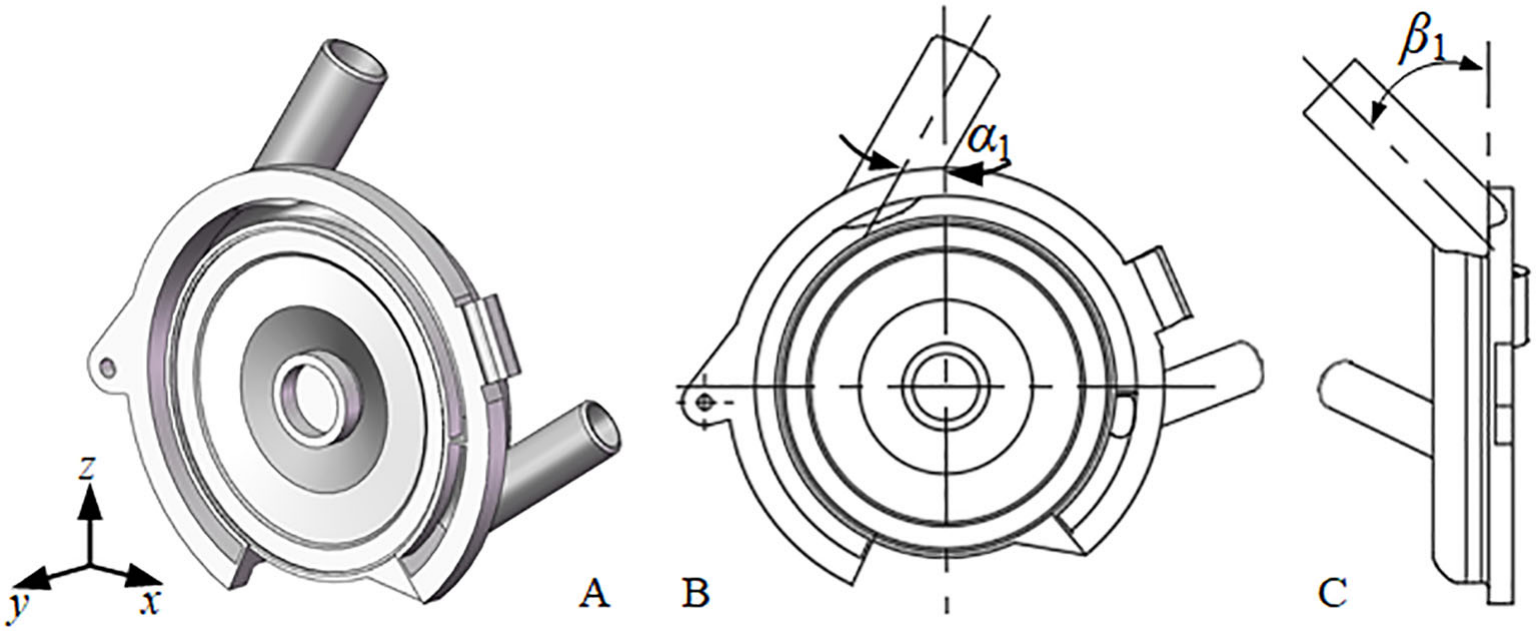
Structural parameter diagram of the negative pressure inlet pipe **(A)** is the axonometric drawing; **(B)** is the xoz plane; **(C)** is the yoz plane.

Based on the investigation of seed dynamics and force conditions during the operation of the seed-metering device, alongside insights from other researchers on air-suction seed-metering devices (Ding et al., 2018; Xu et al., 2022), it is evident that the angle (*α*) between the negative-pressure inlet pipe in the *XOZ* plane and the vertical axis, the angle (*β*) of the inlet pipe in the *YOZ* plane relative to the vertical axis, and the inlet pipe’s taper (*θ*) all significantly impact seeding performance. **Figure 6** presents a schematic representation of these structural parameters. The objective of this research is to identify the optimal structural parameters of the negative pressure inlet pipe by examining its impact on seeding performance through CFD-DEM coupling simulation analysis. Subsequently, the optimal operating conditions for the seed-metering device and the associated seeding performance will be established by evaluating its adaptability to various operational scenarios.

## 3 Materials and methods

### 3.1 CFD-DEM coupling simulation

The interior of the seed-metering device constitutes a standard coupling field comprising both the seed particle field and the airflow field during operation. Therefore, to successfully conduct the simulation test of the coupling process, the following prerequisites must be satisfied: (1) the seed-metering device must be geometrically modeled to facilitate the creation of the airflow field, (2) a particle model of the maize seed must be developed to create the particle field by generating particles via the particle factory, (3) the requisite simulation conditions and parameters must be configured to guarantee the execution of the coupling simulation test, and (4) all coupling simulation tests must adhere to the established testing protocol.

#### 3.1.1 Particle modeling of maize seeds

The “Zhongyu 3” maize variety was selected as the subject of this study. Based on its external dimension characteristics, this variety was categorized into flat and spherical shapes, and a 3D model of the maize seeds was constructed using Solidworks (version 2022, Dassault Systèmes SolidWorks Corporation, Waltham, Massachusetts, United States). Previous studies (Su et al., 2022; Long et al., 2022; Li et al., 2022) have effectively simulated the deformation and fracture behaviors of maize (Kozhar et al., 2015) by employing bonded particle model (BPM) to create maize simulation particles. Consequently, the BPM approach was utilized in this paper to model the imported 3D structure in EDEM 2018 (DEM Solution Ltd., Edinburgh, Scotland), and the API particle replacement method was employed to generate the particle model required for simulation. **Supplementary Figure S1** displays the maize seed particle model, showcasing the particle bonding model, the 3D representation, and the physical image of the seed, arranged sequentially from top to bottom.

#### 3.1.2 Geometry modeling of the seed-metering device

To enhance the computational efficiency of the CFD-DEM coupling simulation, components that did not contribute to the seeding effect were removed. The simplified DEM model of the seed-metering device included the seed inlet, front and rear shells, internal and external cleaning blades, seed layer height adjustment plate, seeding plate, and both positive and negative pressure inlet pipes. The 3D model was finalized in Solidworks, saved in.step format, and imported into EDEM.

Given the intricate internal structure of the seed-metering device, meshing the entire CFD domain presents challenges. It is essential to streamline and modify the structure of the CFD domain without compromising the integrity of key components concerning structural features and seeding efficacy. To accelerate solving efficiency and accuracy, the CFD domain encompassing positive and negative pressure air inlets, holes, individual chambers, a seeding tube, and other elements meshed using a hexahedral structure with the sweeping method. The seeding plate must be rotated around its axis during operation. Accordingly, within the CFD computational domain, the holes should be aligned to rotate per the seeding plate’s rotation in EDEM.

**Figure 7:**
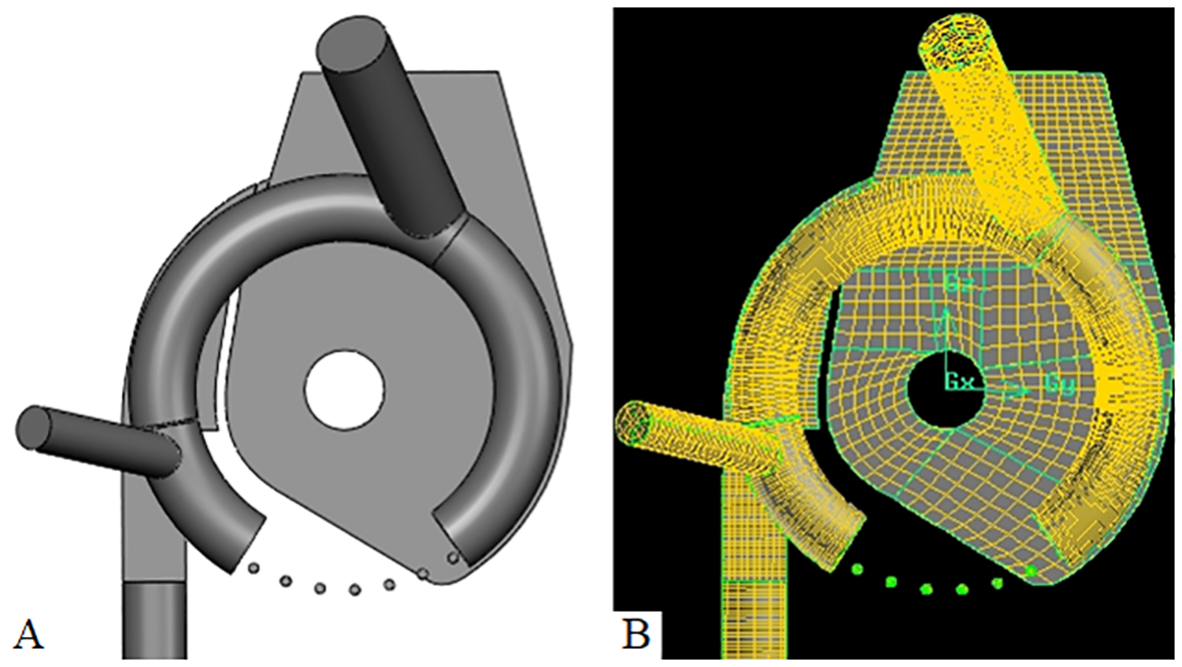
CFD dominated mesh model **(A)** is the CFD domain modeling; **(B)** is the CFD domain meshing.

To replicate the relative rotation between the holes and the internal chamber, the moving mesh technique was employed to construct a rotational model of the holes, enabling real-time monitoring of their spatial positional changes. Simultaneously, the holes were designated as dynamic regions, while the remaining CFD domains were fixed as static regions. The contact surfaces between each component of the CFD domain were configured as interfaces, with the corresponding relationships defined in the solver for data transfer. To ensure the precision of the CFD-DEM coupling simulation, the mesh quality was checked using the Examine Mesh function in Gambit. The proportion of meshes exhibiting skewness between 0 and 0.8 reached 100%, indicating exceptional mesh quality. The CFD domain mesh model is depicted in **Figure 7**.

The seeding plate in EDEM was set to rotate in alignment with the rotation of the holes in the CFD domain, ensuring that both shared the same rotational speed. A particle factory was created within EDEM, utilizing an API particle replacement method to create the maize seed bonded particle model. The simplified DEM model of the seed-metering device and the particle replacement process are depicted in **Supplementary Figure S2**.

#### 3.1.3 Computational conditions and parameters

The simulation parameters are categorized into two groups: material mechanical parameters and material contact parameters, with their values derived from the actual materials used. In this investigation, the seeding plate is constructed from stainless steel, the front shell from transparent Plexiglas, and the remaining components from aluminum alloy. The mechanical properties of maize seeds, stainless steel, Plexiglas, aluminum alloy, and other materials, along with their physical interactions, were utilized to define the physical properties of the seeds and the contact components, as presented in **Table 1**. The Hertz-Mindlin no-slip contact model is based on the contact characteristics of the seeds and the seed-metering device in the coupling tests.

**Table 1:** The input parameters of CFD-DEM coupling simulation.

Type	Parameters	Maize kernel	Organic glass	Aluminum alloy
Solid phase	Poisson’s ratio	0.4	0.50	0.25
	Shear modulus (Pa)	1.37×108	1.77×108	2.70×1010
	Density (kg/m3)	1197	1180	2700
	Restitution coefficient (with maize kernel)	0.182	0.709	0.620
	Static friction coefficient (with maize kernel)	0.0338	0.4590	0.3420
	Rolling friction coefficient (with maize kernel)	0.0021	0.0931	0.0515
	DEM time step (s)	5×10-6		
Gas phase	Fluid	Air		
	Gravitational acceleration (m/s3)	9.81		
	Density (kg/cm3)	1.225		
	Viscosity (kg/m/s)	1.7984×10-5		
	CFD time step (s)	5×10-4		
	Data resources	(Wang et al., 2016; Han et al., 2023; Zhang et al., 2022)		

To accurately simulate the seed-filling process, 74 flat seeds and 26 spherical seeds were generated in proportion to the ungraded seeds. The time step size in EDEM was set to 5×10^-6^ s, while the Fluent time step size was established at 5×10^-4^ s. The number of time steps in Fluent was configured to 6000, resulting in a total simulation time of 3 s. The total simulation duration in EDEM was also set to 3 s, allowing for the rotation of at least 30 holes in the seeding plate at a forward speed of 6 km/h. To capture the motion data of particles in detail, information was recorded in both EDEM and Fluent every 0.02 s.

#### 3.1.4 CFD-DEM coupling procedure

The coupling simulation calculation process is divided into two segments: the airflow field and the particle field. The drag force affects particle motion, while the motion of the particles also interacts with the airflow field, affecting its flow. These elements are interconnected but must be computed separately within two distinct models. Consequently, two separate simulation models for particles and fluids are required. **Supplementary Figure S3** provides a concise overview of the linked flow utilized in this study.

### 3.2 CFD-DEM coupling simulation test scheme

To investigate the influence of the structural parameters of the negative pressure inlet pipe on seeding performance and to identify the optimal combination of specified levels, the structural parameters outlined in section 2.3 were utilized as test factors. These include the angle (*α*) between the negative pressure inlet pipe in the *XOZ* plane and the vertical axis, the angle (*β*) between the inlet pipe in the *YOZ* plane and the vertical axis, and the taper (*θ*) of the inlet pipe. An orthogonal table *L*_9_(3^4^) was chosen to conduct a three-factor, three-level orthogonal experiment. The schematic representations of the test factors and their respective levels are shown in **Supplementary Figure S4**, with the corresponding values detailed in **Table 2**.

**Table 2:** Factors and levels of the orthogonal test.

Levels	Factors		
	*A*, *α* (°)	*B*, *β* (°)	*C*, *θ* (°)
1	0	15	0
2	15	45	2
3	30	75	4

The evaluation indices are significantly affected by the test factors, and the optimal level combinations for these factors at specified levels can be derived from the ANOVA results of the orthogonal test. To further ascertain the optimal combination of the structural parameters of the negative pressure inlet pipe, a central composite test was continued. In this phase, the optimal combination of the test factors identified from the orthogonal test was employed as the central values, and the corresponding level values were calculated. The evaluation indices that were notably affected by the test factors were designated as test indices.

To determine the optimal structural parameters of the negative pressure inlet pipe, a regression model linking the test factors to the evaluation index—referred to as the prediction model—was established through multiple regression analysis of the central composite test results. The prediction model was then solved, with the maximum value of the evaluation index serving as the target value. The solutions derived represent the optimal predicted structural parameters of the negative pressure inlet pipe. Ultimately, the reliability of these optimal structural parameters was validated by comparing the evaluated index values obtained from the coupling simulation tests of the optimized negative pressure inlet pipe with the predicted evaluation index values.

The ANOVA for both the orthogonal and central composite tests was performed using Design-Expert 13 (version 13, Stat-Ease Ltd., Godward St NE, Minneapolis, USA), while the multiple regression analysis for the central composite test results and the resolution of the prediction model for the optimal structural parameters were executed using its Optimization module.

### 3.3 Evaluation index design

Outcome indices such as qualified rate, multiple rate, and leakage rate are utilized to assess seeding performance and are not applicable for evaluating the findings from the coupling simulation tests. Section 3.2 indicates that the coupling simulation is divided into two parts: the airflow field and the particle field. The analysis presented in section 2.2 indicates that in the context of the airflow field, the differential pressure and airflow rate are critical factors influencing seeding performance, necessitating extraction from the Fluent software. Concerning the particle field, the drag force emerges as the primary factor affecting seeding performance, which must be derived from the EDEM software.

#### 3.3.1 Differential pressure

The suction force exerted on seeds is a pivotal aerodynamic parameter that significantly impacts seed filling and transport processes. The seed-metering device proposed in this study is fundamentally reliant on the differential pressure between the negative pressure chamber and the seed-filling compartment to effectively adsorb maize seeds to the seeding plate, thereby underscoring the substantial influence of differential pressure on the seeds’ adsorption efficacy.

Section 2.2 reveals that the adsorption force, represented by the differential pressure during the seed-filling and seed-holding phases, facilitates the stable adhesion of maize seeds to the seeding plate. To enhance the analysis of differential pressure across various operational zones, the holes within the area encompassed by the negative pressure chamber have been systematically numbered, as illustrated in **Supplementary Figure S5**. The differential pressures associated with each working zone were extracted and averaged for both the seed-filling and seed-holding zones, denoted as Δ*p_sf_* and Δ*p_sh_*, respectively.

**Figure 8:**
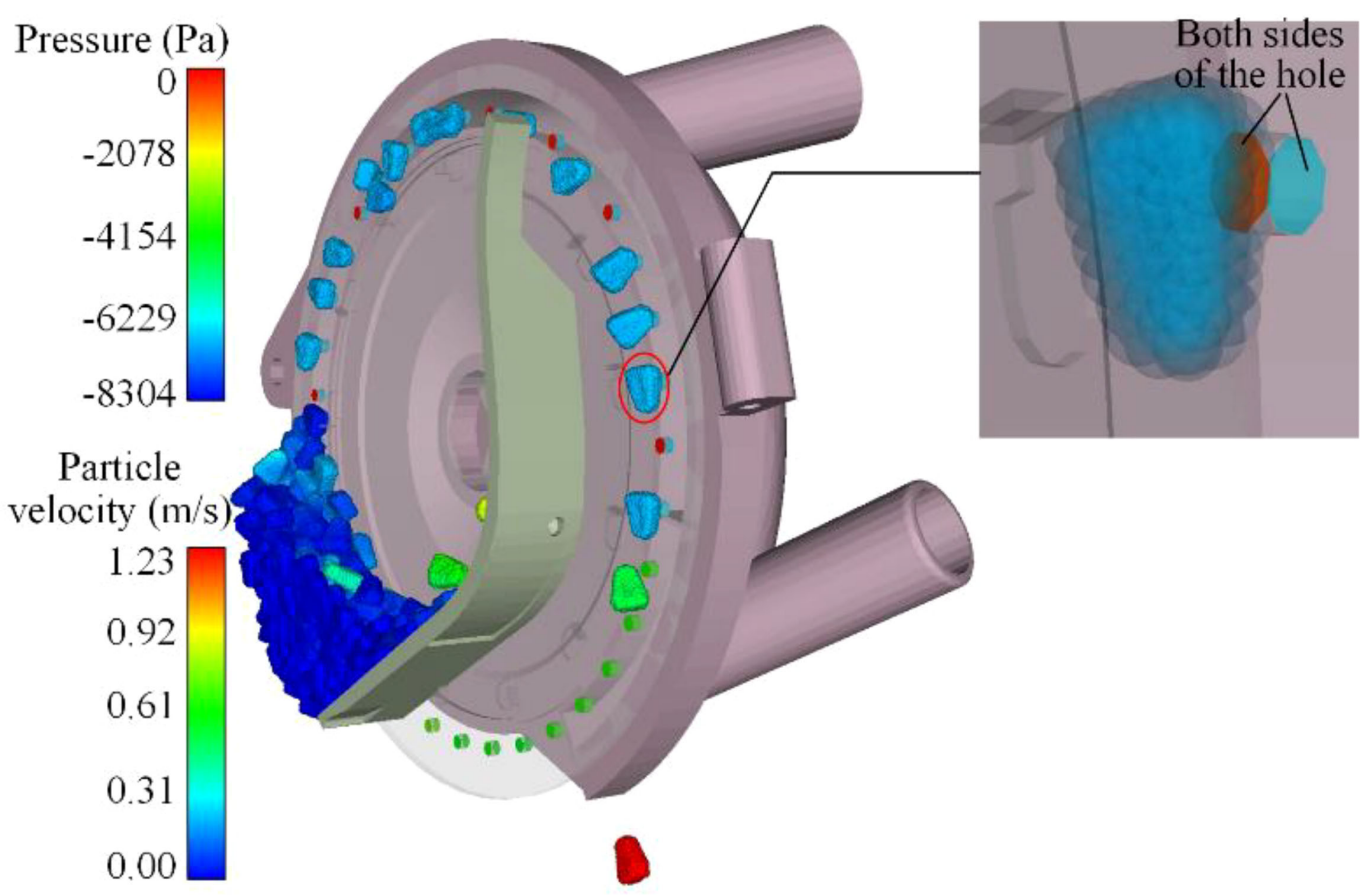
Schematic of pressure extraction position.

**Figure 9:**
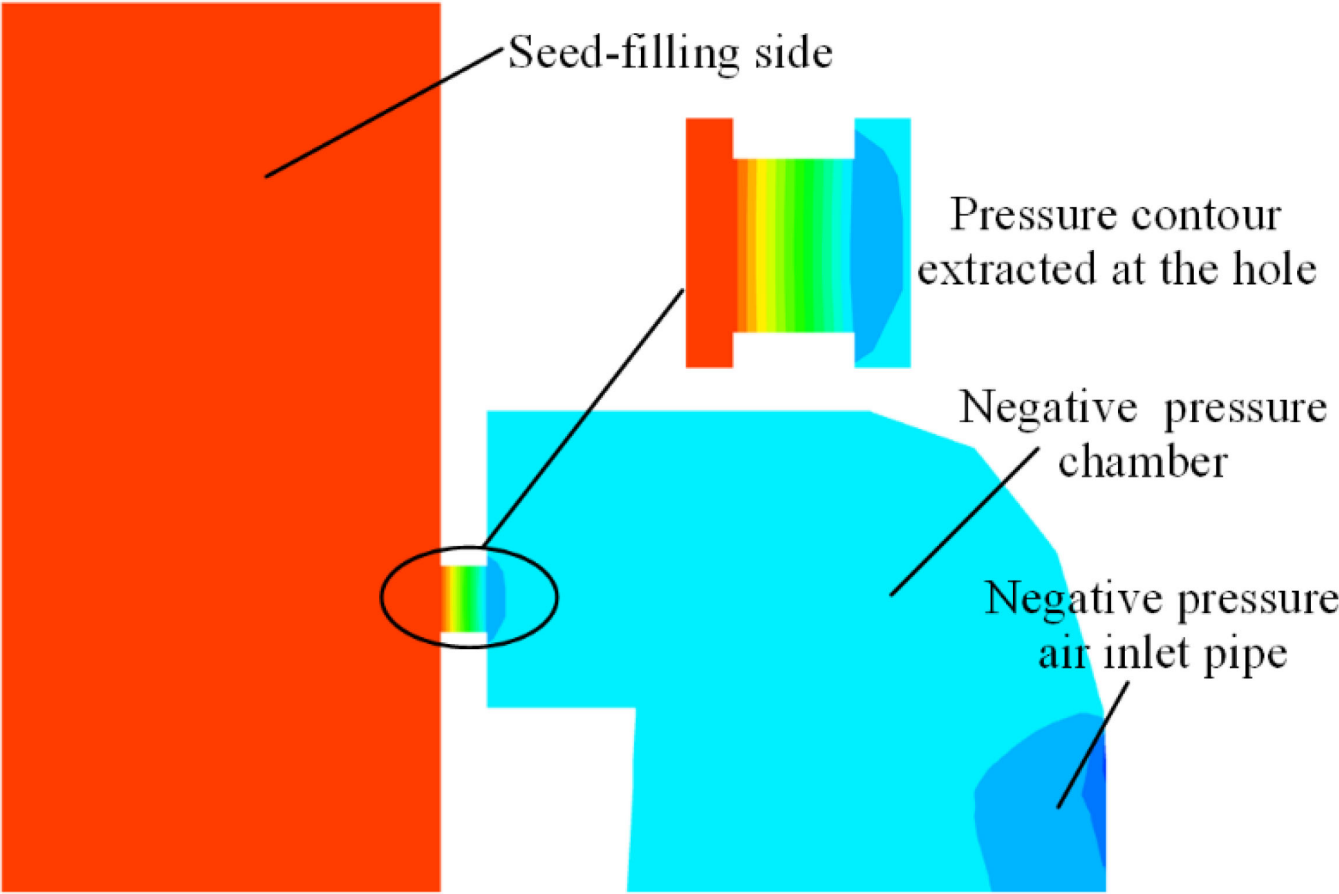
Schematic diagram of the pressure contour extracted location of the hole.

During the meshing of the CFD domain for the seed-metering device, interfaces for each fluid body were established. Consequently, the pressures on either side of each hole were individually post-processed in Fluent before calculating the differential pressure. The locations for pressure extraction are depicted in **Figure 8**. To analyze the pressure variations at the holes within each working zone, pressure contours for the seed-filling and seed-holding zones were obtained. The positions from which these pressure contours were extracted are shown in **Figure 9**. The pressure contours for the holes in each working zone are depicted in **Supplementary Figure S6**.

Given that the negative pressure inlet pipe is situated in proximity to the seed-cleaning zone, the negative pressure within the negative pressure chamber progressively increases in the seed-filling zone, peaks near the seed-cleaning zone, and subsequently declines in the seed-holding zone, as evidenced by the pressure maps for each working zone. The pressure variation at each hole transitions from “0 pa” on the seed-filling side to the maximum negative pressure, indicating that the simulation test is valid and can be utilized for optimization testing.

#### 3.3.2 Airflow rate

In this design, airflow is introduced into the negative pressure chamber from the seed-filling chamber and exits through the outlet. The flow rate (*Q*) at the interface between the negative pressure chamber and the suction pipe greatly influences the overall adsorption seed airflow velocity, thus serving as a crucial evaluation metric for structural optimization. **Supplementary Figure S7** depicts the extraction point for the airflow rate at the interface of the air inlet and the negative pressure chamber.

#### 3.3.3 Drag force

During the operation of the seed-metering device, maize seeds are subjected to a multifaceted environment characterized by airflow, particle, and gravitational forces. The seeds experience a confluence of forces from the airflow field, including drag, buoyancy, and pressure gradients, as well as Basset, Magnus, and Saffman lift forces. Given the relatively low velocity of the seeds, the influence of drag force on the seeds is accentuated, taking into account the unique characteristics of each force. Drag force is defined as the force exerted by a moving airflow on a solid object with a relative velocity. The interaction between the airflow and the seed generates a force. The seed, moving at a lower velocity, exerts a resistance effect on the faster-moving airflow, while simultaneously experiencing a drag force from the airflow (Pasha et al., 2015).

**Figure 10:**
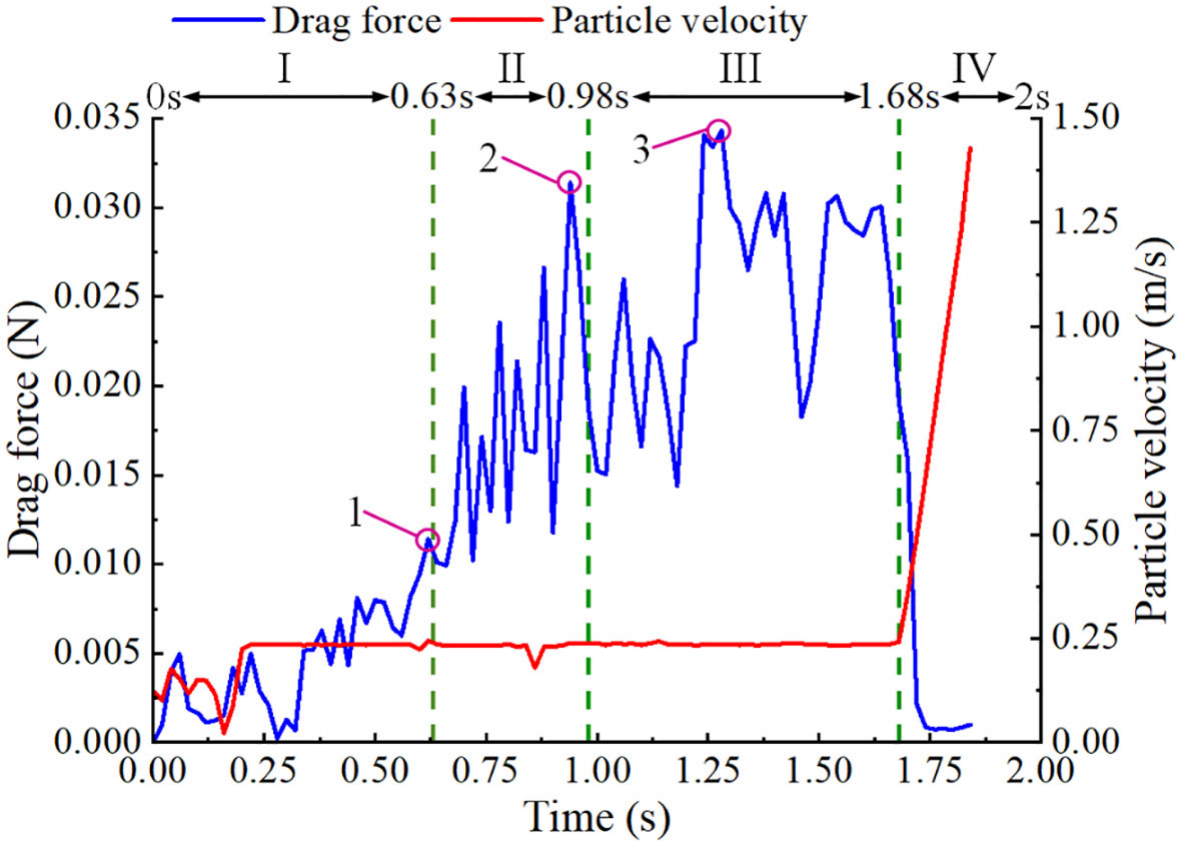
The drag force of seeds in each region. I is the seed-filling zone, II is the seed-cleaning zone, III is the seed-carrying zone, and IV is the seed-voting zone.

An analysis was conducted on the variations in drag force and particle velocity throughout the operational phases of the seed-metering device. The relationships between drag force and particle velocity over time are plotted in **Figure 10**. The drag force exhibited fluctuations as it transitioned from the seed-filling zone to the seed-holding zone, generally trending upwards, before dissipating in the seed-voting zone when the negative pressure airflow was obstructed. During the initial stage in the seed-filling zone, the seeds encountered a complex array of forces as they transitioned from the seed pile to being adhered to the holes, resulting in some variability in particle velocity. Once the seeds were secured by the holes, they maintained a relatively stable state, leading to a consistent particle velocity. As the negative-pressure adsorption force diminished in the seed-voting zone, the seeds descended into the tube, accelerating under the action of gravitational force and positive-pressure auxiliary airflow, which resulted in a rapid increase in particle velocity.

Analysis of the kinematic behavior of the seeds across each working zone, as detailed in section 2.3, revealed that drag force played a crucial role in facilitating the adsorption of maize seeds during the seed-filling, seed-cleaning, and seed-holding processes. The maximum drag forces experienced by the same seed in the seed-filling, seed-cleaning, and seed-holding zones are denoted as 1, 2, and 3 in **Figure 10**, respectively. Following each coupling simulation, the maximum drag forces for ten seeds were extracted from each working zone and averaged, yielding the average drag force in the seed-filling zone (*F*_D,_ *_sf_*), the average drag force in the seed-cleaning zone (*F*_D,_ *_sc_*), and the average drag force in the seed-holding zone (*F*_D,_ *_sh_*).

To thoroughly assess the outcomes of the coupling simulation tests, corresponding evaluation indices were derived from both the airflow and particle fields, as presented in **Table 3**.

**Table 3:** Table of evaluation indexes.

Physical field	Evaluation index	Symbol	Unit
Airflow field in CFD	Averaged differential pressure between the two sides of the holes in the seed-filling zone	Δ*p_sf_*	Pa
	Averaged differential pressure between the two sides of the holes in the seed-holding zone	Δ*p_sh_*	Pa
	The airflow rate in the interaction surface between the negative pressure inlet pipe and the negative pressure chamber	*Q*	m^3^/min
Particle field in EDEM	Averaged drag force on seeds in the seed-filling zone	*F*_D,_ *_sf_*	N
	Averaged drag force on seeds in the seed-cleaning zone	*F*_D,_ *_sc_*	N
	Averaged drag force on seeds in the seed-holding zone	*F*_D,_ *_sh_*	N

### 3.4 The adaptability of the seed-metering device to different working conditions

**Figure 11:**
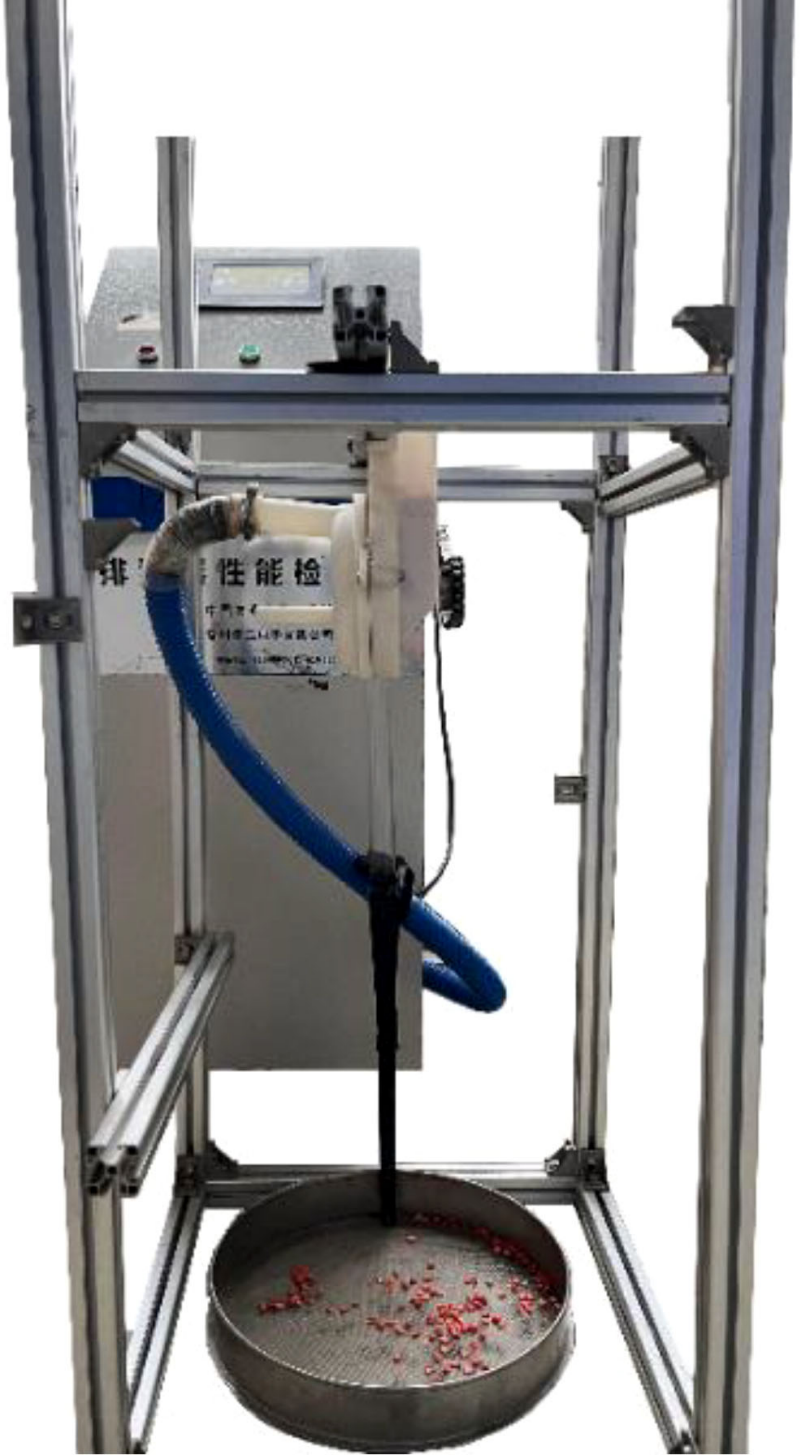
Bench test apparatus.

To ascertain the seeding performance and optimal working conditions of the seed-metering device equipped with the optimized negative pressure inlet pipe under varying working conditions, a bench test is proposed for testing. The rear shell was fabricated and shaped utilizing 3D printing technology, employing the predicted optimal structural parameters of the negative pressure inlet pipe integrated into the seed-metering device. This assembly was subsequently affixed to the PZQCSY-2 seeding performance tester in preparation for the experimental evaluation (**Figure 11**).

Concerning the optimized structural parameters of the negative pressure inlet pipe, an initial comparative bench test was conducted against the pre-optimized air-suction seed-metering device. In this comparative assessment, the theoretical seed spacing was set at 10 cm, the working pressure of the negative pressure inlet pipe was set at -5 kPa, and the working speed of the seeder ranged from 3 to 7 km/h. Furthermore, to achieve a comprehensive understanding of seeding performance under varying operational conditions, a full-factorial test encompassing both working speed and negative pressure will be executed. This will validate the accuracy of the simulation tests and determine the optimal working parameters.

In the full-factorial bench test, the theoretical seed spacing was also established at 10 cm, with the operating speed ranging from 3 to 7 km/h and the operating negative pressure set between 4 to 8 kPa. The maize seed varieties utilized in the experiment were consistent with those employed for particle modeling in section 2.3.1. Following the agronomic requirements for densely planted maize cultivation, the theoretical spacing was set at 10 cm. The evaluation metrics included the qualified rate, multiple rate, and leakage rate. Data collection adhered to the GB/T 6973-2005 (Müller et al., 2006) testing methodologies for single seed drills (precision drills) (National Technical Committee for the Standardization of Agricultural Machinery (2006)), with 250 seeds collected for each trial. Each evaluation index was computed as Equation 7:


(7) $$\left\{ \begin{array}{l} Q=\frac{n_{1}}{N}\times 100\%\\ M=\frac{n_{2}}{N}\times 100\%\\ L=\frac{n_{3}}{N}\times 100\% \end{array}\right.$$

Where *Q* is the qualified rate in %, *M* is the multiple rate in %, *L* is the leakage rate in %, *n*_1_ is the number of single seed holes in one trial, *n*_2_ is the number of holes containing two or more seeds in one trial, *n*_3_ is the count of unseeded holes in one trial, and *N* is the total number of consecutively recorded seeded holes in one trial, with *N* equaling 250.

## 4 Results and analysis

### 4.1 Orthogonal test results and analysis

The extent to which the structural parameters of the negative pressure inlet pipe affect the evaluation indexes can be ascertained from the ANOVA (**Table 4**) of the orthogonal coupling simulation test results presented in **Table 5**, which also reveals the optimal combination of test elements at specified levels.

**Table 4:** Variance analysis of orthogonal test results.

Dependent variables	Sources of variance	Sum of deviation squares	*df*	Mean square	*F*-value	*P*-value	Significance
Δ*p_sf_*	*A*	968535.924	2	484267.962	62.720	0.016	*
	*B*	1044786.352	2	522393.176	67.658	0.015	*
	*C*	3276844.675	2	1638422.338	212.202	0.005	**
	Error	15442.121	2	7721.061			
Δ*p_sh_*	*A*	804077.428	2	402038.714	0.308	0.765	−
	*B*	171479.251	2	85739.625	0.066	0.938	−
	*C*	785110.478	2	392555.239	0.301	0.769	−
	Error	2611332.682	2	1305666.341			
*Q*	*A*	0.025	2	0.012	111.063	0.009	**
	*B*	0.008	2	0.004	34.429	0.028	*
	*C*	0.102	2	0.051	456.438	0.002	**
	Error	0.000	2	0.000			
*F*_D,_ *_sf_*	*A*	5.180E-5	2	2.590E-5	19.958	0.048	*
	*B*	6.836E-6	2	3.418E-6	2.634	0.275	−
	*C*	0.000	2	5.586E-5	43.044	0.023	*
	Error	2.596E-6	2	1.298E-6			
*F*_D,_ *_sc_*	*A*	0.000	2	9.782E-5	177.492	0.006	**
	*B*	3.030E-5	2	1.515E-5	27.492	0.035	*
	*C*	0.000	2	0.000	229.266	0.004	**
	Error	1.102E-6	2	5.511E-7			
*F*_D,_ *_sh_*	*A*	2.274E-5	2	1.137E-5	0.216	0.823	−
	*B*	9.854E-5	2	4.927E-5	0.934	0.517	−
	*C*	0.000	2	0.000	4.404	0.185	−
	Error	0.000	2	5.275E-5			

* means significant influence in a 95% confidence interval, ** means significant influence in a 99% confidence interval, - means no significant influence. The same is below.

**Table 5:** Simulation results of the orthogonal test.

No.	*A*	*B*	*C*	CFD simulation results			DEM simulation results		
				Δ*p_sf_* (Pa)	Δ*p_sh_* (Pa)	*Q* (m^3^/min)	*F*_D,_ *_sf_* (N)	*F*_D,_ *_sc_* (N)	*F*_D,_ *_sh_* (N)
1	1	1	1	4385.95	498.23	1.231	0.0179	0.0274	0.0333
2	1	2	2	3446.78	634.36	1.310	0.0139	0.0298	0.0320
3	1	3	3	3585.59	1650.18	1.063	0.0106	0.0214	0.0270
4	2	1	2	2609.12	1592.01	1.241	0.0184	0.0210	0.0360
5	2	2	3	2167.75	975.84	1.082	0.0109	0.0100	0.0240
6	2	3	1	4387.25	1231.28	1.290	0.0200	0.0250	0.0347
7	3	1	3	2841.20	2011.06	0.901	0.0050	0.0060	0.0090
8	3	2	1	4179.53	2578.07	1.173	0.0131	0.0180	0.0337
9	3	3	2	4010.32	388.12	1.200	0.0137	0.0210	0.0409

From the analysis detailed in **Table 4**, it is evident that the angle (*α*) has a highly significant impact on the airflow rate (*Q*) and *F*_D,_ *_sc_*. Additionally, angle (*α*) also generally influences Δ*p_sf_* and *F*_D,_ *_sf_*. Furthermore, the angle (*β*) demonstrates a generally significant effect on Δ*p_sf_*, the airflow rate (*Q*), and *F*_D,_ *_sc_*. The taper (*θ*) extremely significantly influenced Δ*p_sf_*, the airflow rate (*Q*), and *F*_D,_ *_sc_*, while its impact on *F*_D,_ *_sf_* was also generally notable. Conversely, the effects of the three test factors on Δ*p_sh_* and *F*_D,_ *_sh_* were not statistically significant.

To further elucidate the optimal level combination that substantially affects the evaluation indices of the test factors, a polar analysis of the test results was conducted. **Table 6** presents the outcomes of this analysis after excluding the evaluation indices (Δ*p_sh_*, *F*_D,_ *_sh_*) that were not substantially impacted by the test factors.

**Table 6:** Range analysis of the significant factors influencing evaluation indicators.

Evaluation indicators	Factors	Levels			Extreme Difference	Optimal level	Optimal combination
		1	2	3			
Δ*p_sf_*	*A*	3806.107	3054.707	3677.017	751.40	1	*C*_1_ *A*_1_ *B*_3_
	*B*	3278.757	3264.687	3994.387	729.70	3	
	*C*	4317.577	3355.407	2864.847	1452.73	1	
*Q*	*A*	1.201	1.204	1.091	0.113	2	*C*_2_ *A*_2_ *B*_2_
	*B*	1.124	1.188	1.184	0.064	2	
	*C*	1.231	1.250	1.015	0.235	2	
*F*_D,_ *_sf_*	*A*	0.0141	0.0164	0.0106	0.0058	2	*C*_1_ *A*_2_ *B*_3_
	*B*	0.0138	0.0126	0.0148	0.0021	3	
	*C*	0.0170	0.0153	0.0089	0.0082	1	
*F*_D,_ *_sc_*	*A*	0.0262	0.0164	0.0106	0.0156	1	*A*_1_ *C*_2_ *B*_2_
	*B*	0.0181	0.0229	0.0225	0.0048	2	
	*C*	0.0235	0.0239	0.0125	0.0115	2	

The polar analysis indicates that the test factors affect Δ*p_sf_* in the sequence of *C*>*A*>*B*, with the optimal combination being *C*_1_ *A*_1_ *B*_3_. For *Q*, the order remains *C*>*A*>*B*, with the best combination identified as *C*_2_ *A*_2_ *B*_2_. Regarding *F*_D,_ *_sf_*, the sequence is again *C*>*A*>*B*, with the best combination being *C*_1_ *A*_2_ *B*_3_. In the case of *F*_D,_ *_sc_*, the order shifts to *A*>*C*>*B*, with the optimal combination being *A*_1_ *C*_2_ *B*_2_.

**Table 6** further illustrates that the optimal levels for each test factor varied across the four evaluation indices. Given the substantial impact of these indices on seeding performance, each was assigned a score of 25 points to facilitate the determination of their optimal levels. Additionally, test factors that did not significantly influence the evaluation indicators were excluded from the calculations. The optimal structural parameters for the negative pressure inlet pipe at the specified levels were determined to be 7.5° for the angle (*α*), 55° for the angle (*β*), and 1° for the inlet pipe taper (*θ*).

### 4.2 Response surface test results and analysis

#### 4.2.1 CCD test results and analysis

The coding levels for the central composite test are presented in **Table 7**, while the results of the central composite test and the corresponding ANOVA analysis are shown in **Tables 8**–**10**.

**Table 7:** Factors and levels of central composite design.

Levels	Factors		
	*α* (°)	*β* (°)	*θ* (°)
−1.682	−7.50	25.00	0.00
−1	−1.42	37.16	0.41
0	7.50	55.00	1.00
1	16.42	72.84	1.59
1.682	22.50	85.00	2.00

**Table 8:** Scheme and results of CCD tests.

No.	Factors			CFD simulation results		DEM simulation results	
	*x*_1_	*x*_2_	*x*_3_	Δ*p_sf_* (Pa)	*Q* (m^3^/min)	*F*_D,_ *_sf_* (N)	*F*_D,_ *_sc_* (N)
1	−1	−1	−1	4259.73	1.310	0.0133	0.0209
2	1	−1	−1	4136.09	1.274	0.0152	0.0253
3	−1	1	−1	4934.43	1.313	0.0141	0.0205
4	1	1	−1	4721.32	1.294	0.0171	0.0268
5	−1	−1	1	4376.65	1.318	0.0125	0.0246
6	1	−1	1	3961.30	1.259	0.0114	0.0256
7	−1	1	1	4110.77	1.244	0.0152	0.0268
8	1	1	1	4132.96	1.245	0.0175	0.0267
9	−1.682	0	0	4686.24	1.257	0.0139	0.0282
10	1.682	0	0	4429.35	1.235	0.0168	0.0283
11	0	−1.682	0	4287.63	1.311	0.0141	0.022
12	0	1.682	0	4591.43	1.324	0.0163	0.0281
13	0	0	−1.682	4528.26	1.291	0.0167	0.0203
14	0	0	1.682	4271.96	1.250	0.0133	0.0246
15	0	0	0	4836.68	1.287	0.0135	0.0257
16	0	0	0	4925.34	1.263	0.0141	0.0242
17	0	0	0	5087.42	1.285	0.0152	0.0229
18	0	0	0	4982.35	1.275	0.0155	0.0233
19	0	0	0	5048.23	1.282	0.0149	0.0241
20	0	0	0	4987.39	1.278	0.0146	0.0245

*x*_1_, *x*_2_, and *x*_3_ are the levels of *α*, *β*, and *θ*, respectively.

**Table 9:** Variance analysis of CFD simulation results of the CCD tests.

Sources	*y*_1_ (Δ*p_sf_*)					*y*_2_ (*Q*)				
	Sum of squares	df	*F*-value	*P*-value	Significance	Sum of squares	df	*F*-value	*P*-value	Significance
Model	2.332E+06	9	16.2700	<0.0001	**	0.0121	9	9.8200	0.0007	**
*x*_1_	98860.10	1	6.2100	0.0319	*	0.0016	1	12.0100	0.0061	**
*x*_2_	2.058E+05	1	12.9200	0.0049	**	0.0001	1	0.9935	0.3424	−
*x*_3_	2.646E+05	1	16.6100	0.0022	**	0.0028	1	20.0800	0.0012	**
*x*_1_ *x*_2_	15144.09	1	0.9506	0.3526	–	0.0007	1	5.4000	0.0424	*
*x*_1_ *x*_3_	397.76	1	0.0250	0.8776	–	1.125E-06	1	0.0082	0.9296	−
*x*_2_ *x*_3_	2.292E+05	1	14.3900	0.0035	**	0.0015	1	11.2300	0.0074	**
*x*_1_^2^	4.205E+05	1	26.3900	0.0004	**	0.0016	1	11.7100	0.0065	**
*x*_2_^2^	6.515E+05	1	40.9000	<0.0001	**	0.0031	1	22.7800	0.0008	**
*x*_3_^2^	7.397E+05	1	46.4400	<0.0001	**	0.0001	1	0.3773	0.5528	−
Residual	1.593E+05	10				0.0014	10			
Lack of Fit	1.195E+05	5	3.0100	0.1261	–	0.0010	5	2.6200	0.1574	−
Error	39756.44	5				0.0004	5			
Cor Total	2.492E+06	19				0.0135	19			

* means significant influence in a 95% confidence interval, ** means significant influence in a 99% confidence interval, - means no significant influence.

**Table 10:** Variance analysis of DEM simulation results of the CCD tests.

Sources	*y*_3_ (*F*_D,_ *_sf_*)					*y*_4_ (*F*_D,_ *_sc_*)				
	Sum of squares	df	*F*-value	*P*-value	Significance	Sum of squares	df	*F*-value	*P*-value	Significance
Model	0.0000	9	5.4900	0.0068	**	0.0001	9	5.2200	0.0065	**
*x*_1_	8.823E-06	1	10.7600	0.0083	**	0.0000	1	5.1400	0.0450	*
*x*_2_	0.0000	1	20.6300	0.0011	**	0.0000	1	7.9800	0.0172	*
*x*_3_	5.694E-06	1	6.9400	0.0250	*	0.0000	1	9.8100	0.0069	**
*x*_1_ *x*_2_	2.531E-06	1	3.0900	0.1095	–	8.000E-08	1	0.0406	0.8429	−
*x*_1_ *x*_3_	1.711E-06	1	2.0900	0.1792	–	0.0000	1	6.0900	0.0319	*
*x*_2_ *x*_3_	4.651E-06	1	5.6700	0.0385	*	6.050E-07	1	0.3069	0.5882	−
*x*_1_^2^	1.745E-07	1	0.2128	0.6544	–	0.0000	1	12.5300	0.0048	**
*x*_2_^2^	4.686E-08	1	0.0571	0.8159	–	4.549E-07	1	0.2308	0.6173	−
*x*_3_^2^	2.701E-09	1	0.0033	0.9554	–	6.832E-06	1	3.4700	0.0741	−
Residual	8.202E-06	10				0.0000	10			
Lack of Fit	5.489E-06	5	2.0200	0.2289	–	0.0000	5	3.10	0.1251	−
Error	2.713E-06	5				4.808E-06	5	5.22		
Cor Total	0.0000	19				0.0001	19	5.14		

* means significant influence in a 95% confidence interval, ** means significant influence in a 99% confidence interval, - means no significant influence.

From the ANOVA of the airflow field simulation results in **Table 9**, it is evident that the effects of angle *β* (*x*_2_), taper *θ* (*x*_3_), and their interactions (*x*_2_ *x*_3_), as well as *x*_1_^2^, *x*_2_^2^, and *x*_3_^2^ on Δ*p_sf_* (*y*_1_), were extremely significant. The influence of angle *α* (*x*_1_) was generally significant, whereas the effects of the other factors were not statistically significant. The influence of angle *α* (*x*_1_), taper *θ* (*x*_3_), the interaction (*x*_2_ *x*_3_) between angle *β* (*x*_2_) and taper *θ* (*x*_3_), as well as *x*_1_^2^ and *x*_2_^2^ on the flow rate *Q* (*y*_2_) was found to be highly significant. Conversely, the interaction (*x*_1_ *x*_2_) between angle *α* (*x*_1_) and angle *β* (*x*_2_) exhibited a generally significant effect, while the impacts of other factors were deemed insignificant.

From the ANOVA analysis of the particle field simulation results presented in **Table 10**, it is evident that angle *α* (*x*_1_) and angle *β* (*x*_2_) exerted extremely significant effects on *F*_D,_ *_sf_* (*y*_3_). The taper *θ* (*x*_3_) and the interaction (*x*_2_ *x*_3_) between angle *β* (*x*_2_) and taper *θ* (*x*_3_) demonstrated generally significant effects, whereas the influence of other factors was not significant. The taper *θ* (*x*_3_) and *x*_1_^2^ had extremely significant effects on *F*_D,_ *_sc_* (*y*_4_), while angle *α* (*x*_1_), angle *β* (*x*_2_), and the interaction (*x*_1_ *x*_3_) between angle α (*x*_1_) and taper *θ* (*x*_3_) showed generally significant effects, with other factors being insignificant.

The ANOVA results from the simulation tests concerning airflow and particle fields suggest that the outcomes from the central composite tests can be utilized for developing a predictive model for the structural parameters of the negative pressure inlet pipe.

#### 4.2.2 Comprehensive analysis

**Figure 12:**
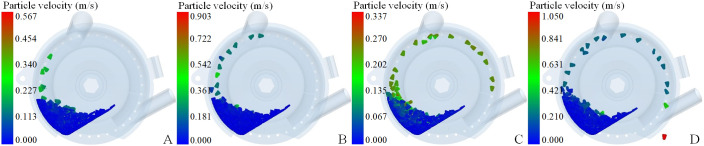
The adsorption state of seeds in each working area **(A)** is seed-filling process; **(B)** is seed-cleaning process; **(C)** is seed-carrying process; **(D)** is seed-voting process.

To enhance the understanding of how the structural characteristics of the negative pressure inlet pipe affect the evaluation index, the CFD-DEM coupling simulation process warrants an assessment. For instance, **Figure 12** depicts the adsorption state of seeds across various working zones, demonstrating that the seeding process generated by the coupling simulation aligns with actual conditions.

**Supplementary Figure S8** presents the fifteen sets of CFD-DEM coupling simulation results derived from the central composite test indicating that the speed and adsorption effects of the seeds differ when they adhere to the seeding plate, influenced by varying structural parameters of the negative pressure inlet pipe. Although tests conducted under identical operating parameters (negative pressure of 7 kPa) yielded a relatively similar degree of seed population boiling and velocity changes within the seed pile, the findings collectively affirm that these test results can inform the establishment of a predictive model for the structural parameters of the negative pressure inlet pipe.

### 4.3 Parameters optimization and validation

To forecast the structural parameters of the negative pressure inlet pipe, the central composite test results in **Table 8** were subjected to multivariate regression analysis. The regression Equation 8 correlating the structural parameters of the negative pressure inlet pipe (*α*, *β*, and *θ*) with the evaluation indices Δ*p_sf_*, *Q*, *F_D,sf_*, and *F_D,sc_* were established accordingly.


(8) $$\left\{ \begin{array}{l} y_{1}=4981.31-85.08x_{1}+122.77x_{2}-139.19x_{3}+43.51x_{1}x_{2}-7.05x_{1}x_{3}-169.27x_{2}x_{3}\\ -170.81x_{1}{\;}^{2}-212.63x_{2}{\;}^{2}-226.56x_{3}{\;}^{2}\\ y_{2}=1.28-0.011x_{1}-0.0032x_{2}-0.0142x_{3}+0.0096x_{1}x_{2}-0.0004x_{1}x_{3}-0.0139x_{2}x_{3}\\ -0.0106x_{1}{\;}^{2}+0.0147x_{2}{\;}^{2}-0.0019x_{3}{\;}^{2}\\ y_{3}=0.0147+0.0008x_{1}+0.0011x_{2}-0.0006x_{3}+0.0006x_{1}x_{2}-0.0005x_{1}x_{3}+0.0008x_{2}x_{3}\\ +0.0001x_{1}{\;}^{2}+0.0001x_{2}{\;}^{2}-0.0000x_{3}{\;}^{2}\\ y_{4}=0.0241+0.0009x_{1}+0.0011x_{2}+0.0013x_{3}+0.0001x_{1}x_{2}-0.0012x_{1}x_{3}+0.0003x_{2}x_{3}\\ +0.0013x_{1}{\;}^{2}+0.0002x_{2}{\;}^{2}-0.0007x_{3}{\;}^{2} \end{array}\right.$$

Where *x*_1_ is the angle (*α*) between the negative pressure inlet pipe at the *XOZ* plane and the vertical axis, *x*_2_ is the angle (*β*) between the inlet pipe at the *YOZ* plane and vertical axis, *x*_3_ is the taper of the inlet pipe (*θ*), *y*_1_ is the average differential pressures across the two sides of the holes in the seed-filling zone (Δ*p_sf_*) in Pa, *y*_2_ is the airflow rate at the interaction interface between the negative pressure inlet pipe and the negative pressure chamber (*Q*) in m^3^/min, *y*_3_ is the average drag force in the seed-filling zone (*F*_D,_ *_sf_*) in N, and *y*_4_ is the average drag force in the seed-cleaning zone (*F*_D,_ *_sc_*) in N.

The ANOVA results presented in **Tables 9**, **10** demonstrate that the prediction models exhibit a high goodness of fit (*p*<0.0068), with the lack of fit terms showing no significant effects (*p*>0.1251). This indicates that the prediction models are suitable for determining the optimal structural parameters of the negative pressure inlet pipe. The optimal parameter solution model for the negative pressure inlet pipe was constructed using the maximum values of the evaluation indices Δ*p_sf_*, *Q*, *F*_D,_ *_sf_*, and *F*_D_ *_,sc_* as target values, while the maximum and minimum levels of the test factors listed in **Table 6** served as constraints.


(9) $$\left\{ \begin{array}{l} \max(y_{1},y_{2},y_{3},y_{4})\\ s.t.\{\begin{array}{l} -7.5{}^{\circ}\leq x_{1}\leq 22.5{}^{\circ}\\ 25{}^{\circ}\leq x_{2}\leq 85{}^{\circ}\\ 0{}^{\circ}\leq x_{3}\leq 2{}^{\circ} \end{array} \end{array}\right.$$

The solution to Equation 9 was obtained using the Optimization module of the Design-Expert software, yielding the predicted optimal parameters for the negative pressure inlet pipe, which are as follows: the angle (*α*) is 17.1°, the angle (*β*) is 81.3°, and the taper of the inlet pipe (*θ*) is 0.52°.

**Table 11** provides a comparison between the predicted values of the evaluation indices and those obtained from the coupling simulation verification test of the seed-metering device equipped with the enhanced negative pressure inlet pipe. The validation test results indicate that Δ*p_sf_* is 4526 Pa, the airflow rate *Q* is 1.26 m^3^/min, *F*_D,_ *_sf_* is 0.0168 N, and *F*_D,_ *_sc_* is 0.029 N. These results are largely consistent with the anticipated theoretical optimal search outcomes, with the maximum error being less than 6.66%, thereby affirming the validity of the prediction model for the structural parameters of the negative pressure inlet pipe.

**Table 11:** Simulation verification test results.

Parameter	Evaluation metrics			
	Δ*p_sf_* (N)	*Q* (m^3^/min)	*F*_D,_ *_sf_* (N)	*F*_D,_ *_sc_* (N)
Predicted value	4651	1.32	0.0180	0.028
Measured value	4526	1.26	0.0168	0.029
Error (%)	2.68	4.55	6.66	3.57

### 4.4 Analysis of the adaptability of the seed-metering device to different working conditions

#### 4.4.1 Improved analysis of the seeding performance with negative pressure inlet pipe

**Figure 13:**
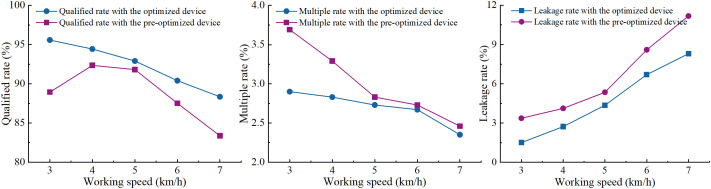
Results of the negative pressure inlet pipe on seeding performance enhancement.

For the structural parameters of the optimized negative pressure inlet pipe, comparative bench tests were conducted alongside the pre-optimized air-suctioin seed-metering device, with results illustrated in **Figure 13**. The qualified rate of the seed-metering device equipped with the optimized negative pressure inlet pipe has improved to a certain extent, while both the multiple rate and leakage rate have been reduced. This indicates that the optimized negative pressure inlet pipe enhances seeding performance. Additionally, as depicted in **Figure 13**, the qualified rate and multiple rate of the seed-metering device with the optimized negative pressure inlet pipe tend to decrease as the working speed of the seeder increase, whereas the leakage rate shows a gradual increase with the rising working speed. Therefore, to optimize the performance metrics of qualified rate, multiple rate and leakage rate, a full factorial test was carried out on the seed-metering device featuring the optimized negative pressure inlet pipe.

#### 4.4.2 Seeding performance under various operating conditions

**Figure 14:**
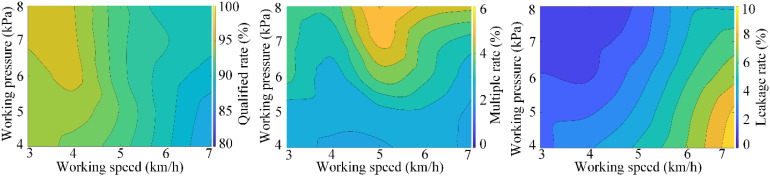
Bench test results.

The results of the adaptation test are displayed in **Figure 14**, showcasing the performance of the seed-metering device with the optimized negative pressure input pipe under varying operational conditions.

As the working speed ranges from 3 to 7 km/h and the working negative pressure varies between 4 to 8 kPa, it is observed that the qualified rate exhibits a gradual decrease and increase in response to the rising working speed and working negative pressure. As the working negative pressure increases, the multiple rate also rises, while the leakage rate tends to increase with the working speed but decreases as the working negative pressure escalates.

Consequently, the optimal operational parameters for the seed-metering device should be set within a working speed range of 4 to 5 km/h and a working negative pressure range of 4 to 6 kPa, considering the energy losses and operational efficiency during field applications. Under these specified conditions, the seed-metering device achieves a qualified rate ranging from 92.27% to 95.28%, a multiple rate between 2.43% and 5.52%, and a leakage rate from 1.22% to 5.2%.

## 5 Conclusions

In this study, a coupling simulation test methodology was employed to investigate the effect of the structural parameters of the negative pressure inlet pipe on the seeding performance of the positive-negative pressure combined seed-metering device. The adaptability of the optimized seed-metering device to varying operational conditions was assessed to identify the optimal working conditions and their corresponding seeding performance, leading to the following conclusions:

1. Orthogonal tests indicate that the structural parameters of the negative pressure inlet pipe significantly affect evaluation metrics such as Δ*p_sf_*, the airflow rate (*Q*), *F*_D,_ *_sf_*, and *F*_D,_ *_sc_*. The evaluation metrics that are notably influenced yield optimal performance when the angle (*α*) is set at 7.5°, the angle (*β*) is 55°, and the taper of the inlet pipe (*θ*) is 1°.
2. Results from the central composite test results demonstrate that the coupling simulation accurately reflects the seeding process of the seed-metering device, and these test outcomes can be utilized to develop a predictive model for the structural parameters of the negative pressure inlet pipe.
3. The predictive model for the structural parameters of the negative pressure inlet pipe exhibits strong goodness of fit, with the impacts of the lack of fit terms being negligible, thereby rendering it suitable for predicting structural parameters. The ideal combination of predicted structural parameters for the negative pressure inlet pipe includes an angle (*α*) of 17.1°, an angle (*β*) of 81.3°, and a taper (*θ*) of 0.52°. The evaluation index values derived from the coupling simulation tests utilizing this optimal parameters combination align closely with the predicted values, exhibiting a maximum deviation of no more than 6.66%, thereby affirming the plausibility of the optimal prediction parameters.
4. The seed-metering device, equipped with the optimized negative pressure inlet pipe, operates most effectively at a speed of 4 to 5 km/h and a negative pressure range of 4 to 6 kPa. Under these operational conditions, the device achieves a qualified rate between 92.27% and 95.28%, a multiple rate from 2.43% to 5.52%, and a leakage rate ranging from 1.22% to 5.2%.

## Data Availability

The original contributions presented in the study are included in the article/**Supplementary Material**. Further inquiries can be directed to the corresponding author/s.

## Supplementary material

Supplementary Figure 1 Modeling of the maize kernel bonded particles.

Supplementary Figure 2 DEM geometrical model.

Supplementary Figure 3 CFD-DEM coupling flowchart.

Supplementary Figure 4 Schematics of the factors and levels for the air inlet position.

Supplementary Figure 5 Schematic diagram of the hole numbering.

Supplementary Figure 6 Pressure contours of the holes in each working zone.

Supplementary Figure 7 Schematic diagram of airflow retrieved position. I is the seed-filling zone, II is the seed-cleaning zone, III is the seed-carrying zone, and IV is the seed-voting zone.

Supplementary Figure 8 Simulation renderings of CCD test 1~15 series.
