# Supplementary figures and images for: CFD-DEM coupling analysis of the negative pressure inlet structural parameters on the performance of integrated positive-negative pressure seed-metering device
Source: Front Plant Sci. 2025 Mar 14;16:1485710. doi: 10.3389/fpls.2025.1485710 (PMC11949883; doi:10.3389/fpls.2025.1485710)

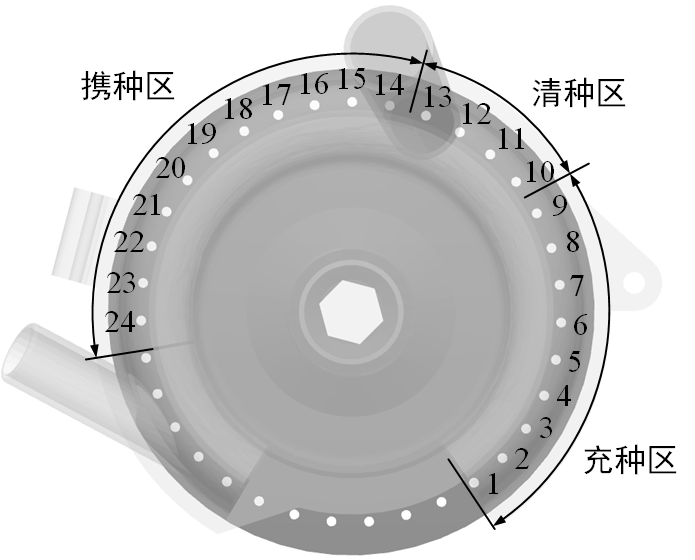

Supplement: Supplementary Figure 1 — Modeling of the maize kernel bonded particles. [file DataSheet1.zip › FIGURE S5 Schematic diagram of the hole numbering.tif]

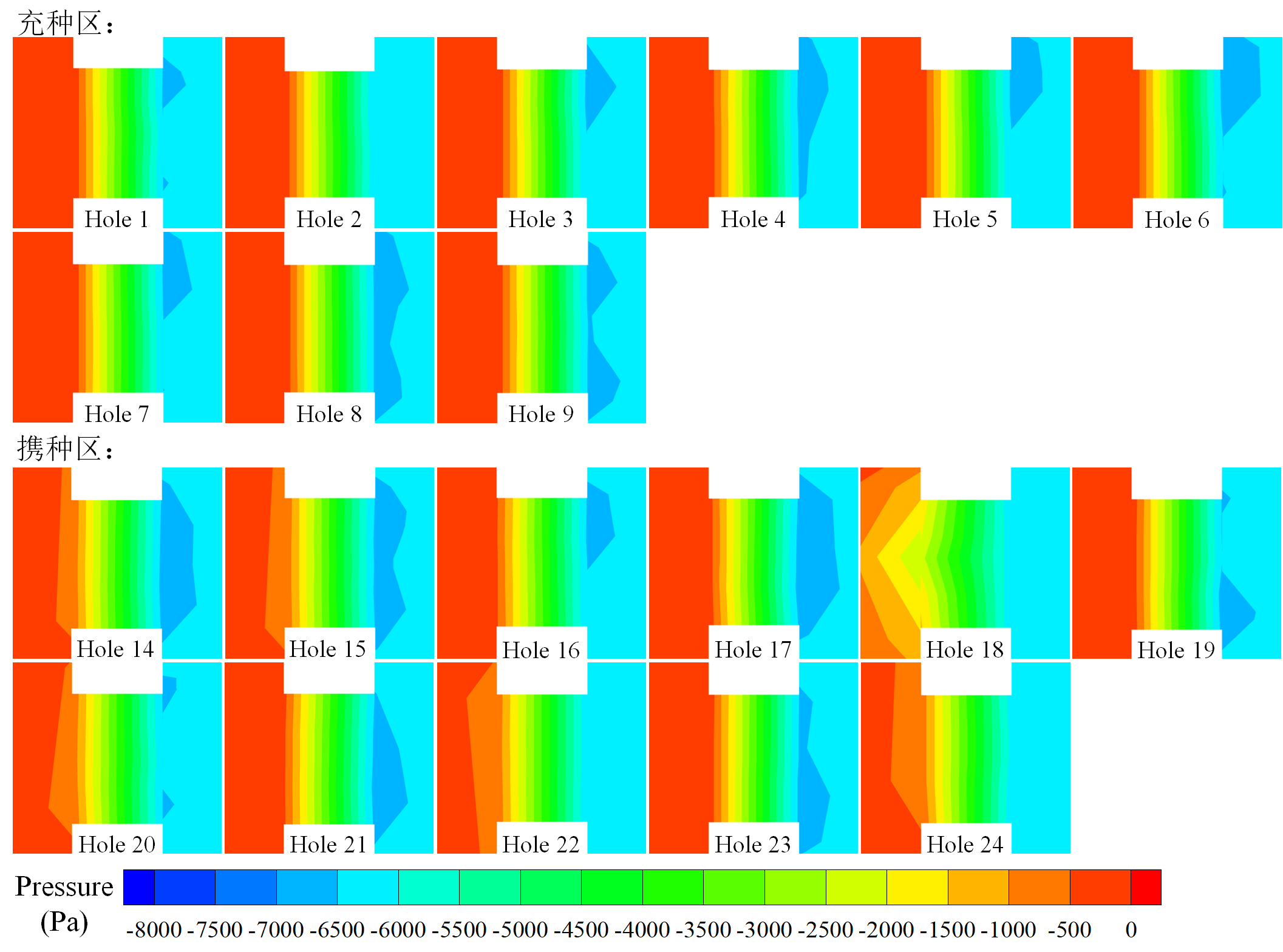

Supplement: Supplementary Figure 1 — Modeling of the maize kernel bonded particles. [file DataSheet1.zip › FIGURE S6 Pressure contours of the holes in each working zone.tif]

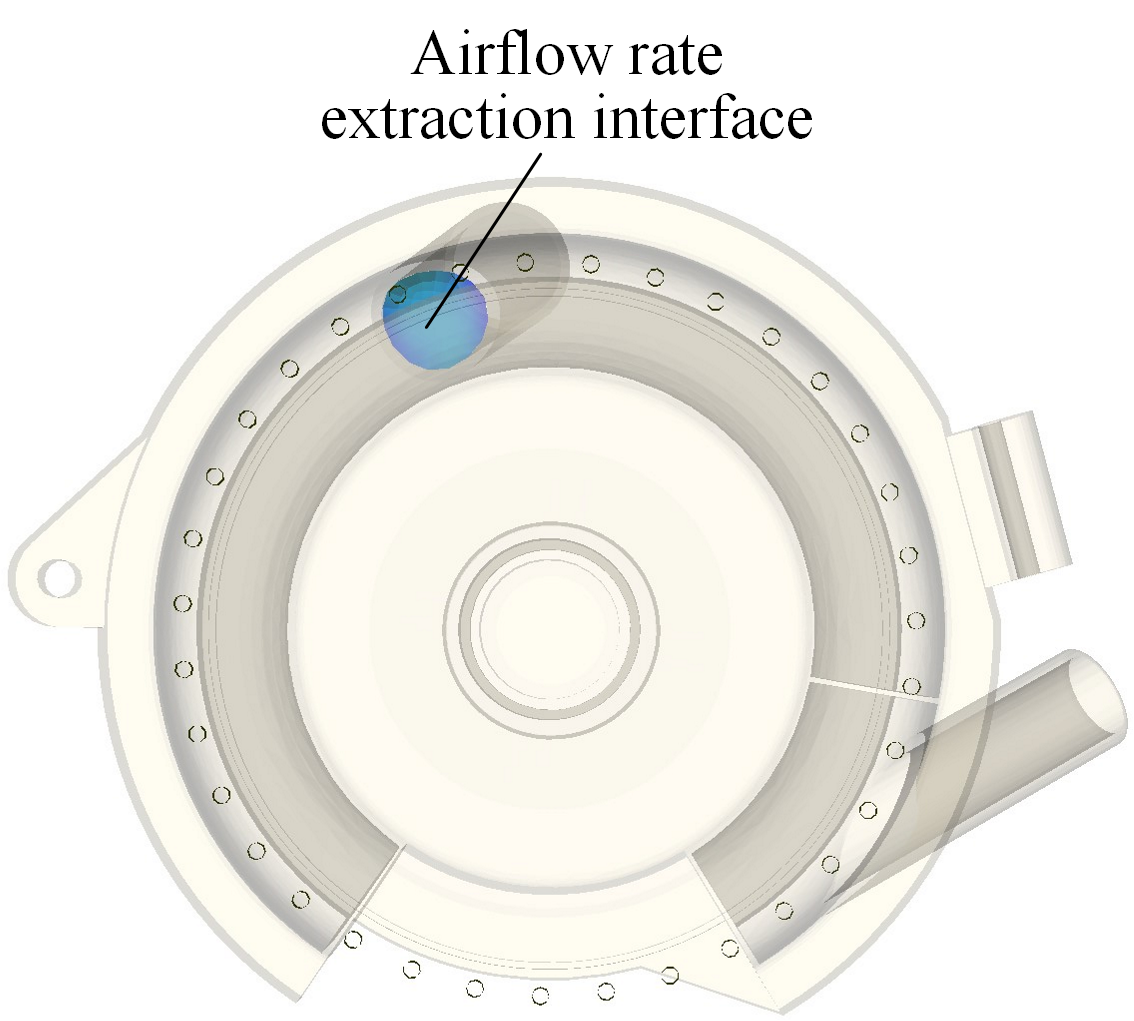

Supplement: Supplementary Figure 1 — Modeling of the maize kernel bonded particles. [file DataSheet1.zip › FIGURE S7 Schematic diagram of airflow retrieved position.tif]

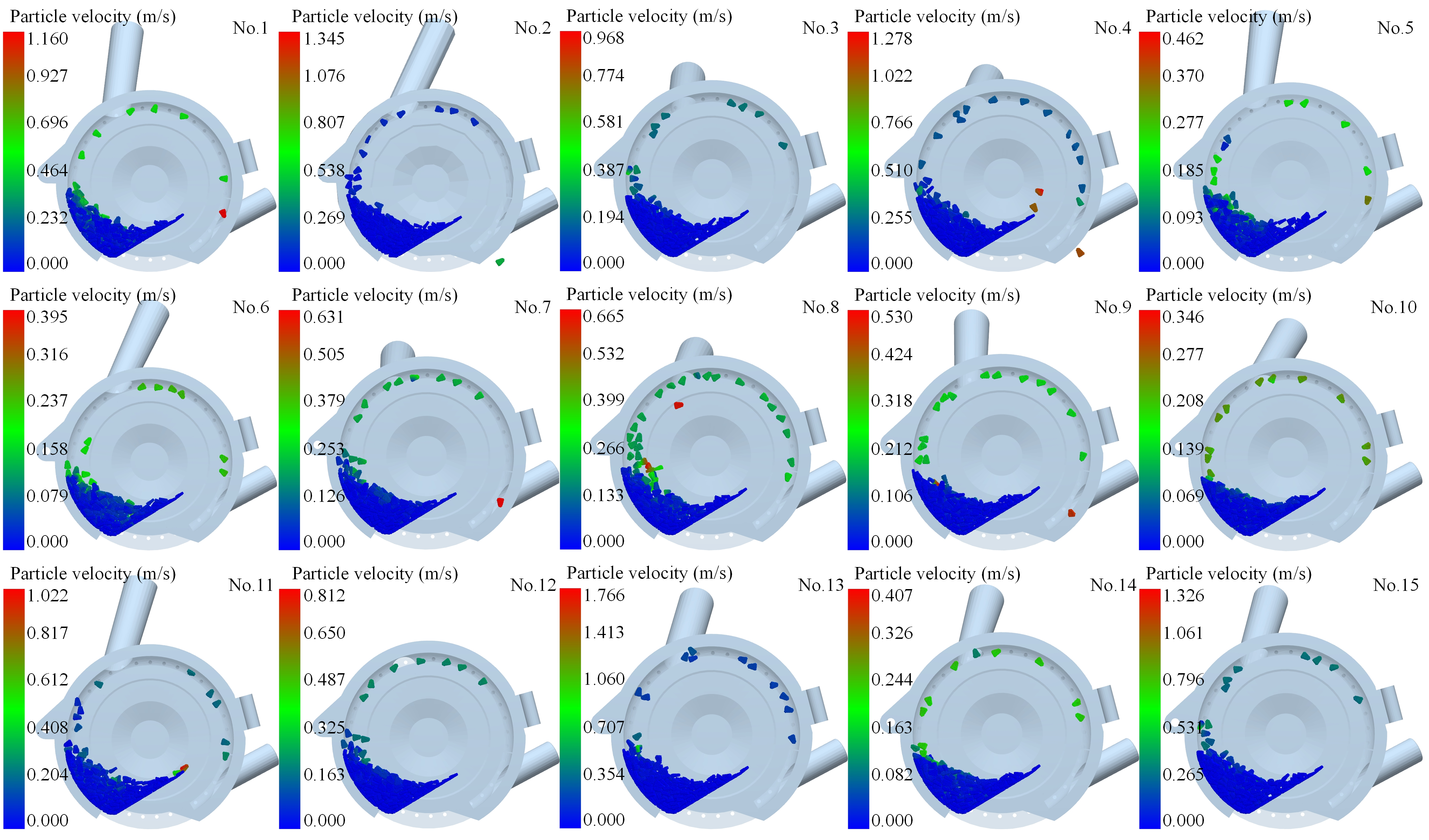

Supplement: Supplementary Figure 1 — Modeling of the maize kernel bonded particles. [file DataSheet1.zip › FIGURE S8 Simulation renderings of CCD test 1~15 series.tif]

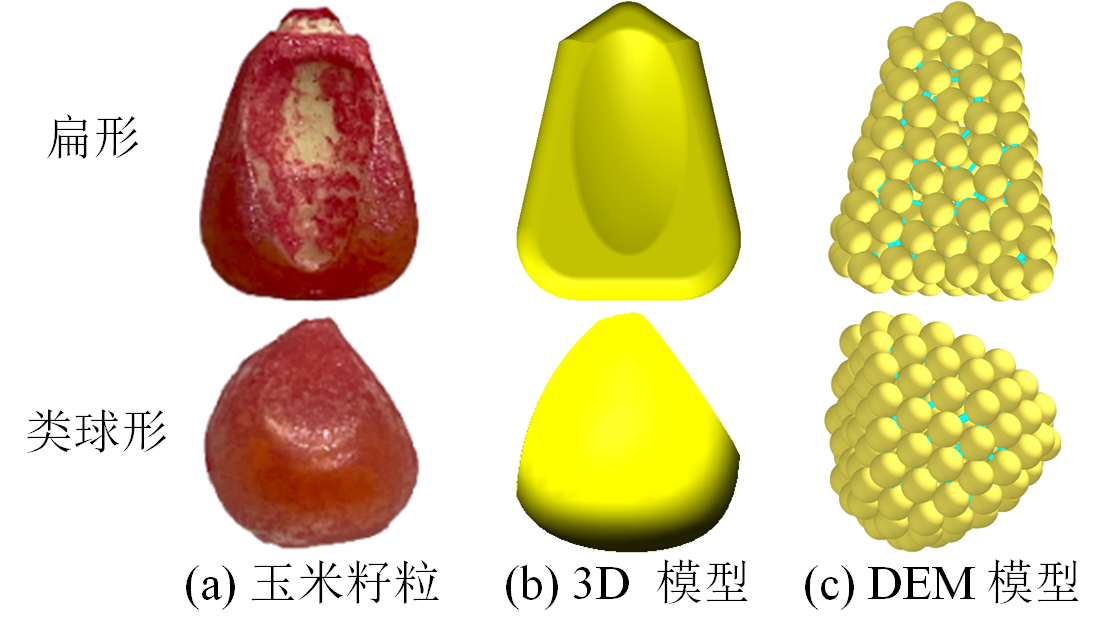

Supplement: Supplementary Figure 1 — Modeling of the maize kernel bonded particles. [file DataSheet1.zip › FIGURE S1 Modeling of the maize kernel bonded particles.tif]

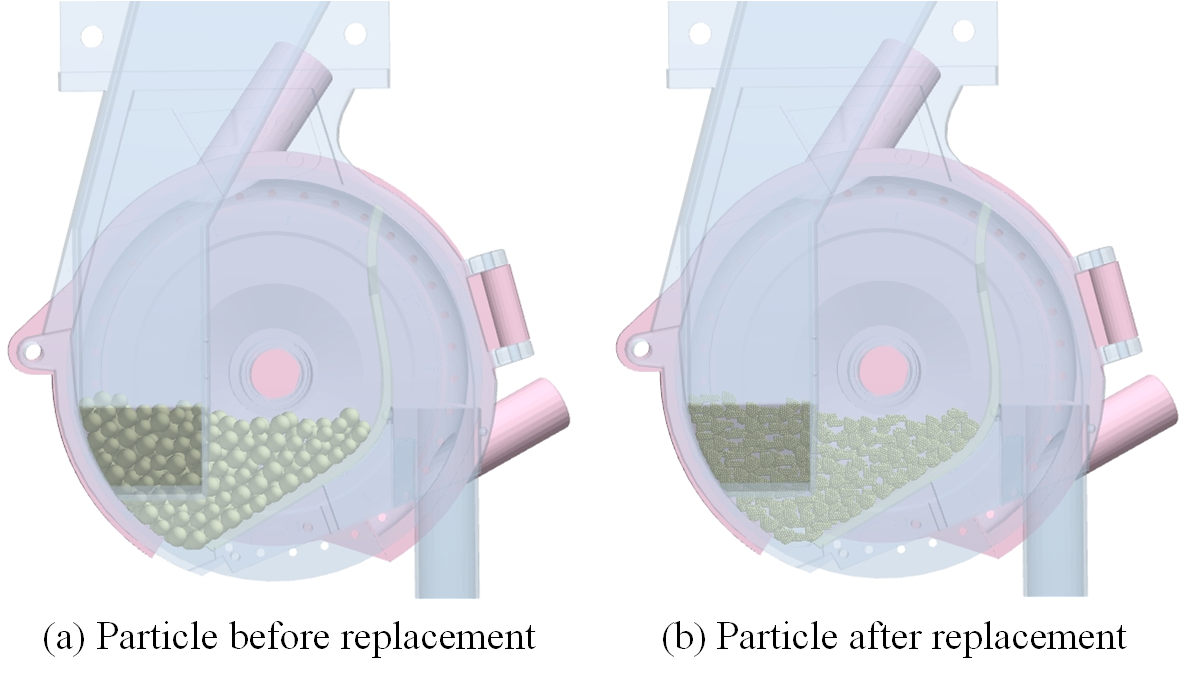

Supplement: Supplementary Figure 1 — Modeling of the maize kernel bonded particles. [file DataSheet1.zip › FIGURE S2 DEM geometrical model.tif]

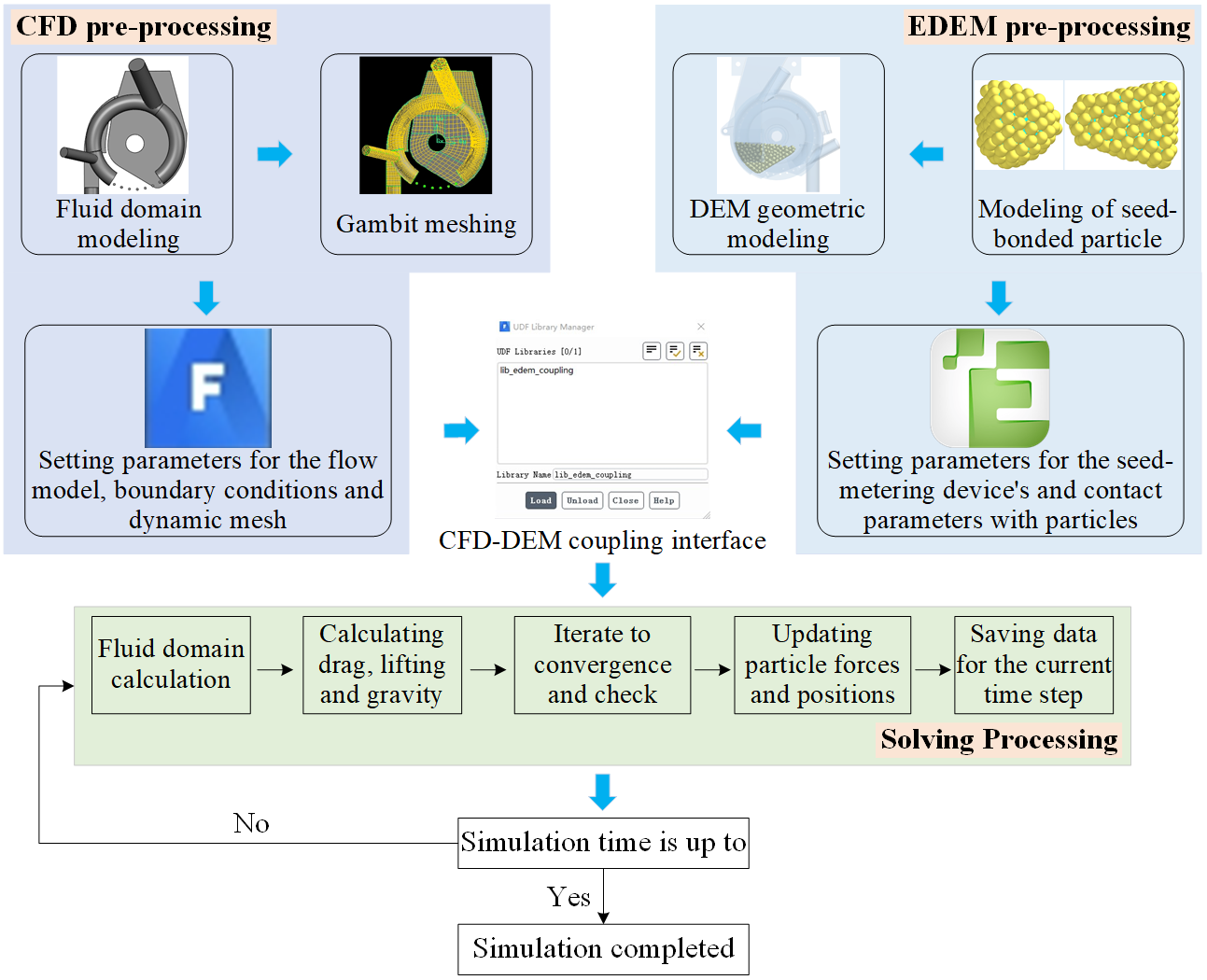

Supplement: Supplementary Figure 1 — Modeling of the maize kernel bonded particles. [file DataSheet1.zip › FIGURE S3 CFD-DEM coupling flowchart.tif]

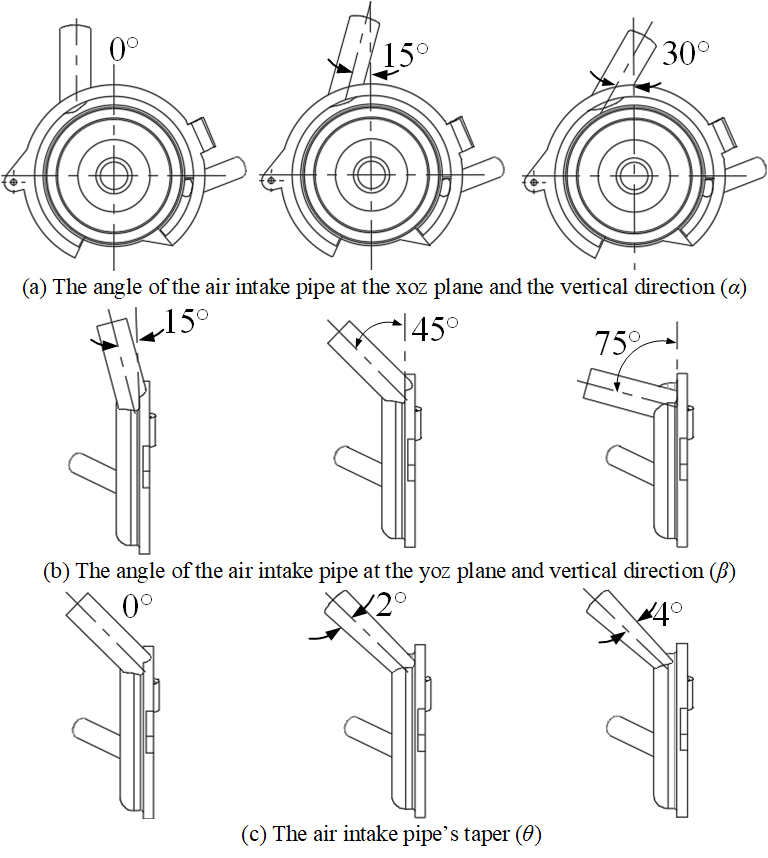

Supplement: Supplementary Figure 1 — Modeling of the maize kernel bonded particles. [file DataSheet1.zip › FIGURE S4 Schematics of the factors and levels for the air inlet position.tif]
